# Unraveling the directional relationship of sleep and migraine-like pain

**DOI:** 10.1093/braincomms/fcae051

**Published:** 2024-02-18

**Authors:** Robson C Lillo Vizin, Caroline M Kopruszinski, Paula M Redman, Hisakatsu Ito, Jill Rau, David W Dodick, Edita Navratilova, Frank Porreca

**Affiliations:** Department of Pharmacology, College of Medicine, University of Arizona, Tucson, AZ 85724, USA; Department of Pharmacology, College of Medicine, University of Arizona, Tucson, AZ 85724, USA; Department of Pharmacology, College of Medicine, University of Arizona, Tucson, AZ 85724, USA; Department of Anesthesiology, University of Toyama, Toyama 930-0194, Japan; Department of Neurology, Bob Bové Neuroscience Institute at HonorHealth, Scottsdale, AZ 85251, USA; Department of Neurology, Mayo Clinic, Phoenix, AZ 85054, USA; Department of Pharmacology, College of Medicine, University of Arizona, Tucson, AZ 85724, USA; Department of Pharmacology, College of Medicine, University of Arizona, Tucson, AZ 85724, USA

**Keywords:** migraine, sleep quality, sleep fragmentation, mice

## Abstract

Migraine and sleep disorders are common co-morbidities. Patients frequently link their sleep to migraine attacks suggesting a potential causal relationship between these conditions. However, whether migraine pain promotes or disrupts sleep or whether sleep disruption can increase the risk of migraine remains unknown. We assessed the potential impact of periorbital allodynia, a measure consistent with migraine-like pain, from multiple preclinical models on sleep quantity and quality. Additionally, we evaluated the possible consequences of sleep deprivation in promoting susceptibility to migraine-like pain. Following the implantation of electroencephalogram/electromyography electrodes to record sleep, mice were treated with either single or repeated systemic injections of nitroglycerin at the onset of their active phase (i.e. nocturnal awake period). Neither single nor repeated nitroglycerin affected the total sleep time, non-rapid eye movement sleep, rapid eye movement sleep, sleep depth or other measures of sleep architecture. To account for the possible disruptive effects of the surgical implantation of electroencephalogram/electromyography electrodes, we used immobility recordings as a non-invasive method for assessing sleep-wake behaviour. Neither single nor repeated nitroglycerin administration during either the mouse sleep (i.e. daylight) or active (i.e. night) periods influenced immobility-defined sleep time. Administration of an inflammatory mediator mixture onto the dura mater at either sleep or active phases also did not affect immobility-defined sleep time. Additionally, inhalational umbellulone-induced migraine-like pain in restraint-stressed primed mice did not alter immobility-defined sleep time. The possible influence of sleep disruption on susceptibility to migraine-like pain was evaluated by depriving female mice of sleep over 6 h with novel objects, a method that does not increase circulating stress hormones. Migraine-like pain was not observed following acute sleep deprivation. However, in sleep-deprived mice, subthreshold doses of systemic nitroglycerin or dural calcitonin gene-related peptide induced periorbital cutaneous allodynia consistent with migraine-like pain. Our data reveal that while migraine-like pain does not significantly disrupt sleep, sleep disruption increases vulnerability to migraine-like pain suggesting that a therapeutic strategy focused on improving sleep may diminish migraine attacks.

## Teaser

Lillo Vizin *et al*. report a directional relationship between sleep and migraine-like pain: while migraine-like pain does not disrupt the sleep of male and female mice, sleep disruption increases vulnerability to migraine-like pain. The findings suggest that a therapeutic strategy focused on improving sleep may diminish the likelihood of migraine attacks.

## Graphical Abstract

**Graphical Abstract:**
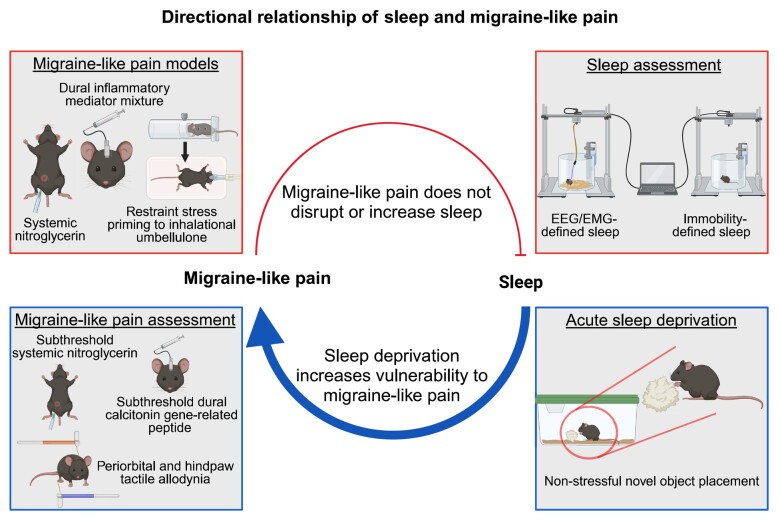

## Introduction

Sleep disorders are commonly reported in people with migraine.^1^ Patients with migraine report insomnia,^2^ trouble falling or staying asleep,^3^ poor sleep quality,^4-7^ excessive daytime sleepiness,^8-10^ waking up from sleep and being forced to sleep or choosing to sleep because of migraine headache.^3,11^ Chronic migraine patients report worse sleep quality,^6^ increased fatigue severity^12^ and shorter nightly sleep duration than those with episodic migraine. Chronic migraine patients are also more likely to report trouble falling or staying asleep.^3,11^ Conversely, disrupted sleep, too much sleep, not enough sleep and lack of sleep routine have commonly been suggested by patients to be triggers that promote migraine.^13,14^ The nature and possible causal direction of interactions of migraine and sleep therefore remain uncertain.

Most conclusions about the relationship between sleep and migraine have been based on subjective reports from patients using questionnaires. Alterations in sleep quantity or sleep macrostructure of migraine patients, however, are not well established with objective assessments of sleep.^6,15-18^ We therefore aimed to unravel (i) the possible causal relationship between migraine headache-like pain and sleep and (ii) the consequences of sleep deprivation on susceptibility to migraine-like pain. We used multiple preclinical models of migraine and assessed sleep both with implanted EEG/EMG recordings and non-invasive immobility measures. Sleep deprivation was induced without increasing circulating markers of stress to determine possible increased responses to subthreshold stimuli that could serve as migraine ‘triggers’. Our data reveal a strong influence of sleep deprivation on susceptibility of migraine-like pain but do not indicate that migraine-like pain enhances or disrupts sleep.

## Materials and methods

### Animals

Female and male C57BL6/J mice (9- to 10-week-old) from the Jackson Laboratory (Sacramento, CA, USA) were used in this study. Housing conditions consisted of a 12-h light/dark cycle (lights on at 7 a.m.) in a climate- and humidity-controlled environment with food and water provided *ad libitum* in the University of Arizona animal facility. All experimental procedures were performed following the ARRIVE guidelines, the ethical guidelines of the International Association for the Study of Pain regulations on animal welfare and the National Institutes of Health guidelines for the care and use of laboratory animals. The experimental procedures were previously approved by the Institutional Animal Care and Use Committee of the University of Arizona. Every effort was made to minimize the number of animals and their suffering. Animals were randomly assigned to their conditions/treatments. Treatments were performed in a blind fashion.

### Drugs

Nitroglycerin (NTG; American Regent, Shirley, NY, USA) was diluted in saline to concentrations of 0.01 and 1 mg/mL and administered intraperitoneally (i.p.) at 0.1 (subthreshold dose) and 10 mg/kg, respectively. Caffeine and doxepin (Sigma, St. Louis, MO, USA) were diluted in saline to concentrations of 2 and 1.5 mg/mL and administered at 20 and 15 mg/kg, respectively. Controls received saline. All intraperitoneal injections were administered at a 10 mL/kg volume. Doses of NTG,^19-22^ caffeine^23^ and doxepin^24^ were chosen based on previous reports. Umbellulone (UMB; AdipoGen, San Diego, CA, USA) was prepared as a stock solution of 0.1 M in 100% dimethyl sulfoxide and freshly diluted to 0.01 M with phosphate-buffered saline for delivery by inhalation.^25^ Inflammatory mediator (IM) mixture consisted of bradykinin (1 mM), histamine (1 mM), 5HT (1 mM) and PGE2 (100 µM) diluted in synthetic interstitial fluid (SIF), pH 4.0.^26^ Controls received SIF, which contained 10 mM HEPES, 5 mM KCl, 135 mM NaCl, 1 mM MgCl2, 2 mM CaCl2 and 10 mM glucose, pH 7.4.^26,27^ All components for IM and SIF were from Sigma. Calcitonin gene-related peptide (CGRP; Bachem, Torrance, CA, USA) stock solution was prepared in ddH_2_O (10 pg/μL) and diluted in SIF to be administered at a subthreshold dose of 0.1 pg.^27^ IM, CGRP or SIF (5 μL) were administered by dural injection (see below).

### Dural injection

Dural injections of IM, CGRP or SIF were performed as previously reported.^26^ Dural injectors were modified from commercially available cannulas (Plastic One/Invivo1, part #C313I/SPC, Internal Cannula, Standard, 28 gauge). The projection length of the injector was adjusted, using a stopper, to 0.65 mm for female and 0.7 mm for male mice, which allows dural administration while maintaining dura mater integrity. The injectors were connected to a 25 μL Hamilton syringe via a Tygon tubing (Cole-Parmer Co, Vernon Hills, IL, USA). For each injection, mice were briefly anaesthetized with 5% isoflurane and the injector was inserted through the skin and the skull at the junction of the sagittal and lambdoid sutures. Then, 5 μL of IM, CGRP or SIF was delivered onto the dura mater. Dural injectors were replaced every four animals.

### Periorbital and hindpaw frequency of response evaluation

Periorbital and hindpaw frequency of response to tactile stimulation was performed as previously reported.^25^ Mice were acclimated in clear plexiglass chambers (10 cm L × 10 cm W × 20 cm H), on top of a wire mesh stand (0.635 cm² grid), for 2 h. After habituation, 0.4 and 0.6 g von Frey filaments (Stoelting Co, Wood Dale, IL, USA) were applied 10 times to the periorbital and hindpaw region, respectively. The filament was gently applied until the filament was slightly arched. Responses were scored after each filament application. Periorbital responses were characterized by facial grooming, head shaking and/or turning away after filament application. Hindpaw responses were characterized by sharp withdrawal of the paw, shaking and/or licking the paw. Cutaneous allodynia was defined by the increased frequency of response to tactile stimulation. Frequency of response was calculated as [number of positive responses/10 × 100%] (i).Frequency of response as a change from baseline was calculated as [frequency of response at test - frequency of response at baseline] (ii).

### EEG/EMG head mount surgery

Under 2–5% isoflurane anaesthesia, mice were implanted with EEG/EMG electrodes for polysomnographic recordings (Pinnacle Technology, Lawrence, KS, USA) as described previously.^28^ Briefly, mice were mounted in a stereotaxic head holder and a 2 cm incision was made down the midline from just behind the eyes to expose the cranium. Dedicated 2 EEG/1 EMG mouse head mounts (Pinnacle Technology) were implanted with four EEG electrode screws. This was accomplished by drilling four holes with avoidance of visible blood vessels to allow the insertion of electrode screws at 1 mm anterior from bregma and lambda, both 1.5 mm lateral to the midline. Two Teflon-coated stainless steel wires were placed bilaterally into both trapezius muscles to record EMG. The head mount was secured to the skull with two anchor screws and covered by dental acrylic. Mice were allowed to recover from the surgery for at least 7 days before recording.

### EEG/EMG recording and analysis

Mice were individually placed in clear plexiglass sleep chamber (241 mm diameter) allocated in a dedicated quiet room and allowed to acclimate to the experimental conditions for at least 3 days. EEG and EMG were recorded through a preamplifier and cable with a low-torque commutator, which allowed the mice unencumbered freedom of movement. Data were collected using Sirenia software (Pinnacle Technology). All data were taken from the EEG electrodes positioned on the frontal lobe. Data were analysed by in a blinded fashion by SleepSign software (Kissei Comtec, Matsumoto, Nagano, Japan). The vigilance state of every 5-s epoch was automatically classified into wake, rapid eye movement (REM) or non-REM (NREM) sleep. Wakefulness was defined by a high EMG amplitude and a low EEG amplitude; REM sleep was defined by a low EMG amplitude, low EEG amplitude and high theta (*θ*) wave (5.0–10.0 Hz) activity; and NREM sleep was defined by a low EMG amplitude, high EEG amplitude and high delta (*δ*) wave (0.65–4.5 Hz) activity. The normalized power spectrum in each frequency during NREM sleep was calculated by dividing the EEG power (μV^2^) of each frequency band by the sum of the EEG power (μV^2^) of all frequencies and expressed as a percentage. As a final step, defined sleep–wake stages were examined visually and corrected, if necessary.

### Immobility-defined sleep recording and analysis

Mice were individually placed in the same sleep chamber used for EEG/EMG recording in a dedicated quiet room and allowed to acclimate to the experimental conditions for at least 2 days. Mice were recorded using a camera (model 9022; Pinnacle Technology), mounted above each chamber, and Sirenia software (Pinnacle Technology). Video recordings were exported, edited using VSDC Video Editor to include a background and analysed in a blinded fashion using ANY-maze (Version 7.0, Stoelting Co). Immobile episodes were defined as lasting more than 40 s at 95% sensitivity detection.^29^ Immobility-defined sleep was quantified by the duration of immobile episodes during 1-h epochs in a 24-h cycle.^29^

### RS priming

Repeated restraint stress (RS) priming was performed according to Kopruszinski *et al*.^25^ Briefly, mice were single-placed in plastic restrainers (551-BSRR; Plas Labs Inc., Lansing, MI, USA). The tail was threaded through the restraint stopper and adjusted to limit the movements without restricting respiration. Mice underwent RS for 2 h each day for 3 consecutive days.

### UMB inhalation exposure

Inhalational exposure to UMB was performed as previously described.^25^ A multi-station isoflurane anaesthesia board (Parkland Scientific, Coral Springs, FL, USA) was used with medical-grade oxygen. A half-square gauze was placed in each nose cone of the anaesthesia board, and 500 µL of 0.01 M UMB (or phosphate-buffered saline, for controls) was pipetted onto each gauze. RS-primed mice were individually placed in each nose cone under light isoflurane anaesthesia (1.5–2%), with exposure to UMB or phosphate-buffered saline for 30 min. Heating pads were placed under the mice to avoid hypothermia, and mice were continuously monitored during the entire procedure.

### Acute sleep deprivation

Sleep deprivation was performed as previously described by Alexandre *et al*.^23^ Briefly, female mice were single housed and sleep deprived for 6 h, starting at light onset (7:00 a.m.), by providing new nesting material or novel objects in their home cage, tapping the cage only when necessary. Different novel objects were provided without touching the mouse when the mouse was idle for more than 1 min. Mice were not disturbed when they were spontaneously awake. The first item provided was always nesting material. Additional toys were designed to incite chewing, a non-stressful innate behavior.^30^ Sham mice were subjected to similar conditions but left undisturbed.

### Mouse corticosterone and prolactin ELISA

Immediately after acute sleep deprivation, mice were anaesthetized with 2–5% isoflurane and whole blood was collected by inferior vena cava puncture and coagulated at room temperature for 1 h. The serum was isolated by centrifugation at 6000 rcf, 10 min, at 4°C, and stored at −80°C until use. Serum corticosterone was quantified by a mouse corticosterone ELISA kit according to manufacturer instructions (ADI-900-097; Enzo Life Science, Farmingdale, NY, USA). Serum prolactin level was quantified by a mouse prolactin ELISA kit according to the manufacturer’s instructions (ab100736; Abcam, Waltham, MA, USA).

### Statistical analysis

Data were analysed by two-way repeated-measures ANOVA followed by Sidak’s or Tukey’s test as appropriate. Analysis of agreement and correlation between EEG/EMG- and immobility-defined sleep were performed using the Bland–Altman statistical method and Pearson's correlation coefficient, respectively, at a 1-h interval over a 24-h cycle. Statistical analyses were performed using GraphPad Prism 9 (GraphPad Software, La Jolla, CA, USA). Data are presented as mean ± SEM and statistical significance was set at *P* < 0.05. The experimental design can be found in the Supplementary material.

## Results

### NTG-induced migraine-like pain did not disrupt sleep as assessed by EEG/EMG recordings, in female mice

**Figure 1:**
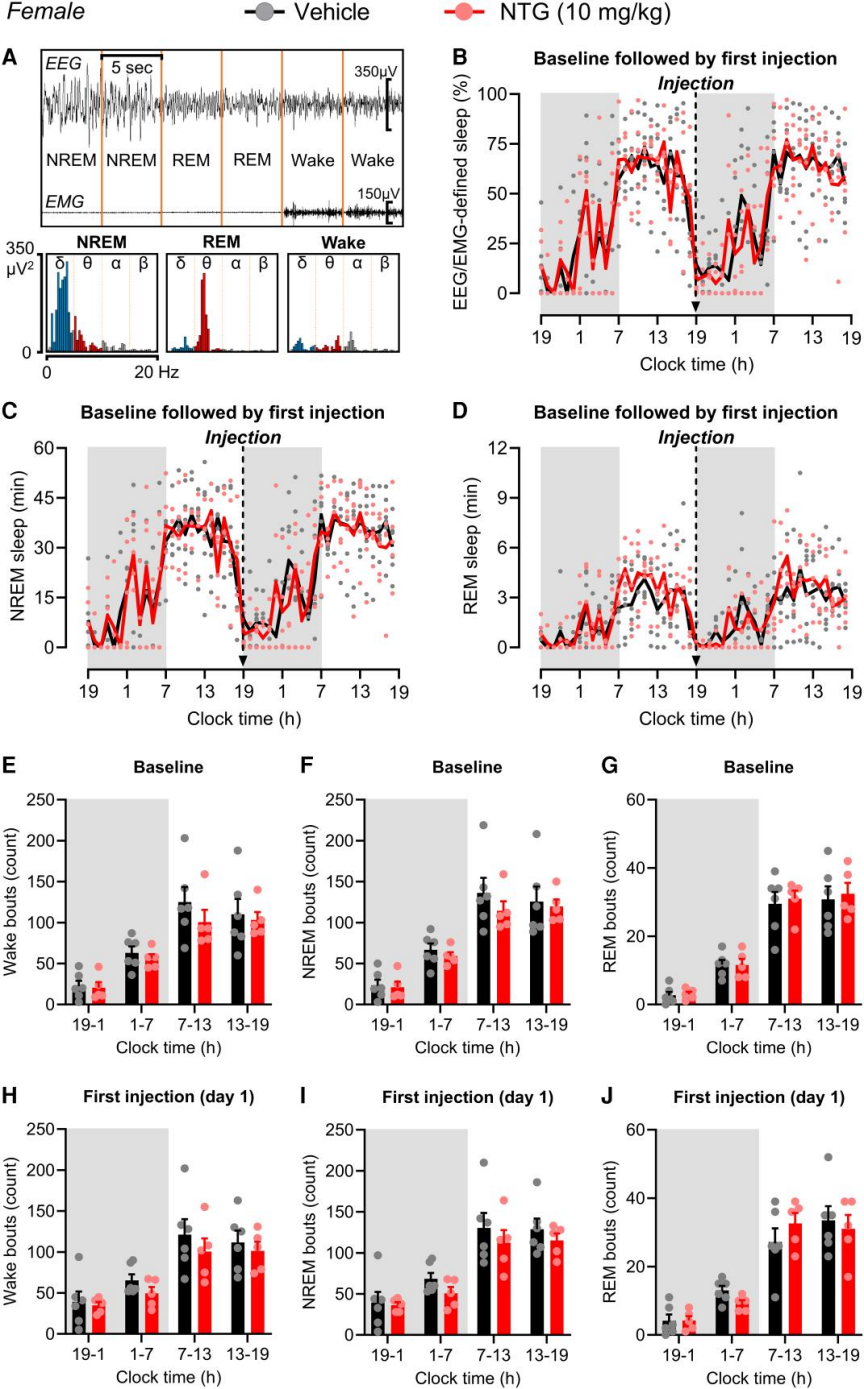
**NTG-induced acute migraine-like pain does not affect the sleep quantity or sleep architecture of female mice.** (**A**) Representative EEG/EMG recording and classification of the sleep/wake vigilance states. The collected EEG/EMG data were analysed, and each 5-s epoch was automatically classified into (i) wakefulness, defined by a high EMG amplitude and low EEG amplitude; (ii) REM sleep, defined by a low EMG amplitude, low EEG amplitude and high *θ* wave (5.0–10.0 Hz) activity; and (iii) NREM sleep, defined by a low EMG amplitude, high EEG amplitude and high *δ* wave (0.65–4.5 Hz) activity. Histograms show the power of *δ* (0.65–4.5 Hz), *θ* (5.0–10.0 Hz) and other waveforms during each vigilance state. (**B–J**) Female mice were implanted with an EEG/EMG head mount to record multiple sleep measures. (**B**) Total sleep, (**C**) NREM and (**D**) REM sleep time were recorded for a 24-h cycle before (baseline) and immediately after a single systemic injection of NTG (10 mg/kg, i.p.) or vehicle at 7 p.m. to induce acute migraine-like pain. Total sleep time is expressed in the percentage of total time in 1-h bins over a 24-h cycle. Wake, NREM and REM bouts are expressed in 6-h bins (early/late light and dark phases) over a 24-h cycle and were recorded at (**E–G**) baseline and (**H–J**) immediately after the first NTG or vehicle injection. Arrows indicate the times of injections. The dark phase is shaded in grey. Data were analysed by two-way repeated-measures ANOVA. (**B**) *F*(47, 423) = 0.6719, *P* = 0.9529. (**C**) *F*(47, 423) = 0.6493, *P* = 0.9652. (**D**) *F*(47, 423) = 0.8449, *P* = 0.7575. (**E**) *F*(3, 27) = 0.4159, *P* = 0.7430. (**F**) *F*(3, 27) = 0.3423, *P* = 0.7949. (**G**) *F*(3, 27) = 0.0625, *P* = 0.9792. (**H**) *F*(3, 27) = 0.2126, *P* = 0.8868. (**I**) *F*(3, 27) = 0.2176, *P* = 0.8833. (**J**) *F*(3, 27) = 1.120, *P* = 0.3582. *F*- and *P*-values are shown for interaction factor (treatment and time). Data values for individual mice are shown as small symbols; lines represent the group means; bars represent the means ± SEM; *n* = 6 for vehicle, and *n* = 5 for NTG. EEG, electroencephalogram; EMG, electromyography; NREM, non-rapid eye movement; NTG, nitroglycerin; REM, rapid eye movement.

Systemic administration of NTG has consistently been shown to induce periorbital and hindpaw allodynia consistent with migraine-like pain,^19,20^ which was confirmed in our experiments (Supplementary Fig. 1A and B). Mice were implanted with an EEG/EMG head mount and randomly divided into vehicle and NTG groups. Figure 1A shows the classification of the sleep/wake vigilance states. Baseline sleep patterns were recorded for a 24-h cycle before vehicle/NTG treatment (Fig. 1B). Female mice exhibited a typical wake/sleep cycle and sleep architecture at baseline, spending most of their time awake (∼81%) during the dark phase and asleep (∼62%) during the light phase (Fig. 1B). Also, typical of the mouse sleep pattern were bouts of wake that occurred during sleep and brief sleep periods during their awake phase (i.e. napping). Mice then received a single injection of either vehicle or NTG (10 mg/kg) at 7 p.m., i.e. immediately before the dark (i.e. active) phase in rodents. This time was chosen to represent the mouse ‘morning’ period as patients often experience migraine headaches in the morning,^31^ and some patients report that migraine attacks cause them to acutely need to sleep.^3^ NTG-induced acute migraine-like pain did not affect the amount of sleep (Fig. 1B and Supplementary Fig. 2A). No significant differences were observed between the groups during baseline or after a single injection of NTG-induced acute migraine-like pain in NREM sleep (Fig. 1C and Supplementary Fig. 2B) or in REM sleep (Fig. 1D and Supplementary Fig. 2C). Additionally, both NTG and vehicle groups showed similar numbers of wake, NREM and REM sleep bouts during baseline (Fig. 1E–G) and after a single injection of NTG (Fig. 1H–J). Further, the mean duration of wake, NREM sleep and REM sleep during baseline (Supplementary Fig. 3A–C) and after a single NTG/vehicle injection (Supplementary Fig. 3D–F) was not different between groups. The power density of delta waves during NREM sleep is closely associated with sleep depth.^28,32,33^ As expected, mice exhibited increased delta-wave power density during baseline NREM sleep in both the dark and light phases (Supplementary Fig. 3G and H), which was not affected by acute NTG treatment (Supplementary Fig. 3I and J). Taken together, these findings indicate that female mice with acute NTG-induced migraine-like pain, administered immediately before the dark phase, maintain a normal sleep/wake cycle and sleep architecture.

**Figure 2:**
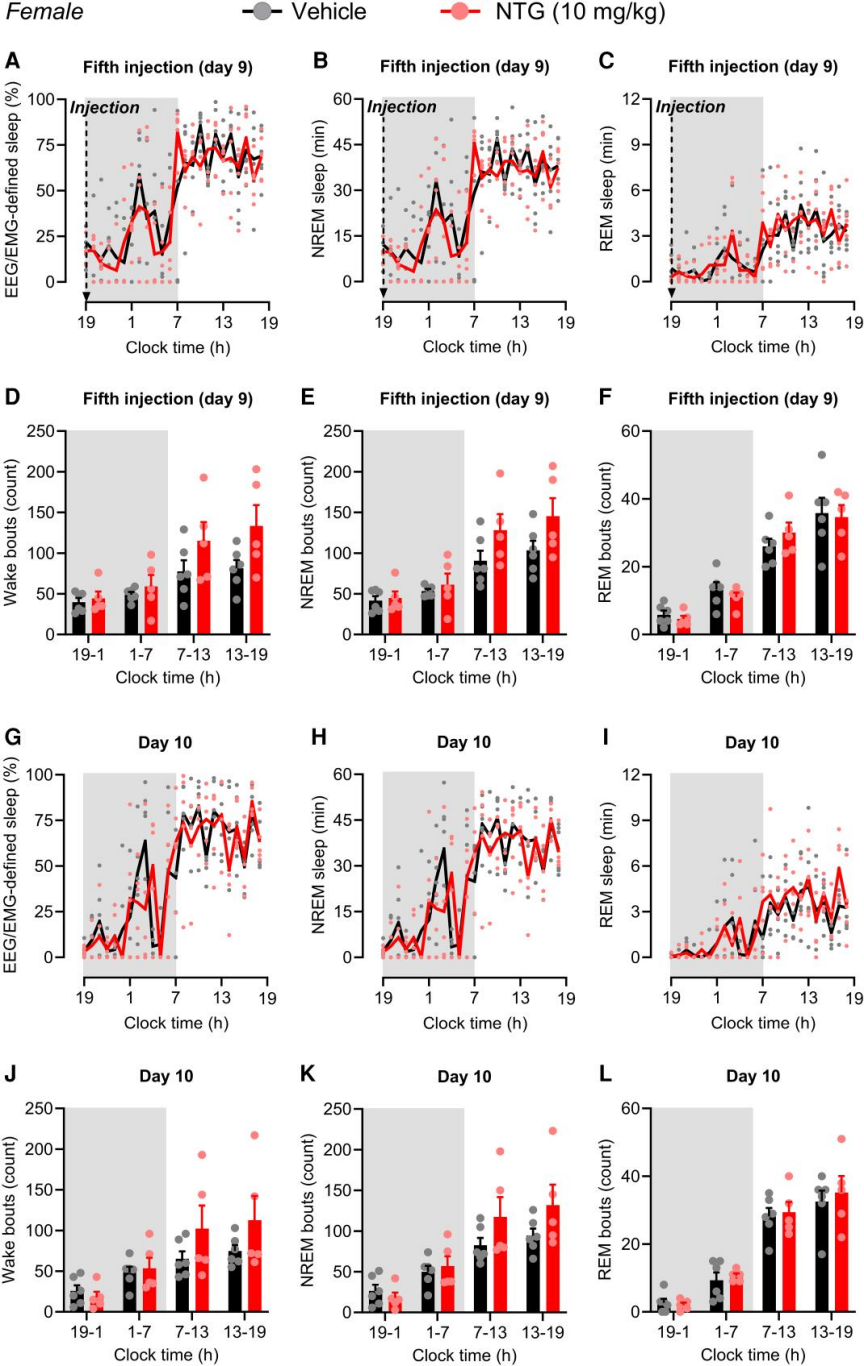
**NTG-induced chronic migraine-like pain does not affect the sleep architecture of female mice.** Female mice were treated with NTG (10 mg/kg, i.p.) or vehicle every other day for 9 days (five injections in total) at 7 p.m. to model a chronic migraine-like pain. Total sleep, NREM and REM sleep time were measured (**A–C**) immediately after the fifth injection (Day 9) and (**G–I**) the following day (Day 10) using EEG/EMG recordings. Total sleep time is expressed in the percentage of total time in 1-h bins over a 24-h cycle. Wake, NREM and REM bouts are expressed in 6-h bins (early/late light and dark phases) over a 24-h cycle and were recorded (**D–F**) immediately after the fifth injection (Day 9) and (**J–L**) the following day (Day 10). Arrows indicate the times of injections. The dark phase is shaded in grey. Data were analysed by two-way repeated-measures ANOVA. (**A**) *F*(23, 207) = 0.8445, *P* = 0.6724. (**B**) *F*(23, 207) = 0.7952, *P* = 0.7354. (**C**) *F*(23, 207) = 0.9653, *P* = 0.5117. (**D**) *F*(3, 27) = 3.690, *P* = 0.0240. (**E**) *F*(3, 27) = 3.189, *P* = 0.0396. (**F**) *F*(3, 27) = 0.7271, *P* = 0.5447. (**G**) *F*(23, 207) = 1.570, *P* = 0.0529. (**H**) *F*(23, 207) = 1.563, *P* = 0.0545. (**I**) *F*(23, 207) = 1.086, *P* = 0.3632. (**J**) *F*(3, 27) = 2.710, *P* = 0.0648. (**K**) *F*(3, 27) = 3.052, *P* = 0.0455. (**L**) *F*(3, 27) = 0.2241, *P* = 0.8788. *F*- and *P*-values are shown for interaction factor (treatment and time). Data values for individual mice are shown as small symbols; lines represent the group means; bars represent the means ± SEM; *n* = 6 for vehicle, and *n* = 5 for NTG. EEG, electroencephalogram; EMG, electromyography; NREM, non-rapid eye movement; NTG, nitroglycerin; REM, rapid-eye movement.

To model chronic migraine-like pain, we continued treating the same female mice with NTG (10 mg/kg) every other day for 9 days (five treatments in total)^19,20^ immediately before the dark (i.e. active) phase. EEG/EMG recordings were then performed for 24-h cycle immediately after the last (fifth) injection (Day 9) and the following day (Day 10). On Day 9, chronic migraine-like pain did not change the amount of total sleep, NREM sleep or REM (Fig. 2A–C and Supplementary Fig. 4A–C) nor the number of wake episodes, NREM or REM bouts (Fig. 2D–F). Similarly, on Day 10, the amount of total sleep, NREM sleep and REM sleep (Fig. 2G–I and Supplementary Fig. 4D–F) of female mice did not differ between NTG and vehicle groups. The number of wake episodes, NREM and REM was also not different between groups (Fig. 2J–L). Further, the mean duration of wake, NREM and REM was not affected by NTG treatment on either Day 9 (Supplementary Fig. 5A–C) or Day 10 (Supplementary Fig. 5D–F). The depth of sleep (power density of delta waves) during the dark or light phase was not affected by chronic NTG treatment on either Day 9 (Supplementary Fig. 5G and H) or Day 10 (Supplementary Fig. 5I and J). Eighteen days after NTG or vehicle treatment onset, i.e. 9 days after the last NTG/vehicle injection, when the effects of NTG-induced pain are resolved,^19^ female mice were treated at the beginning of the dark period with doxepin, a first-generation histamine H_1_R antagonist that induces sleep,^24^ as a positive control. As expected,^24^ doxepin induced sleepiness as indicated by an increase in the percentage of total sleep and NREM sleep (Supplementary Fig. 6A and B). REM sleep was unaffected (Supplementary Fig. 6C).

### Immobility-defined sleep correlates with EEG-defined sleep

Immobility-defined sleep is a non-invasive high-throughput method to evaluate sleep/wake behaviour in mice.^29^ To validate immobility-defined sleep, we compared the immobile time, obtained by video recording, with EEG/EMG-defined sleep in male naïve mice and in response to caffeine or doxepin treatments. Both video and EEG/EMG recordings were captured simultaneously in the same animals. In naïve mice, we observed no difference between EEG/EMG-defined and immobility-defined sleep with both showing similar sleep-time pattern over a 24-h cycle (Supplementary Fig. 7A) with a correlation of 99% (Supplementary Fig. 7B). We also performed a Bland–Altman analysis to evaluate the agreement between EEG/EMG-defined and immobility-defined sleep (Supplementary Fig. 7C). Immobility-defined sleep showed a high agreement with EEG/EMG-defined sleep with a Bland–Altman estimated bias across the 24-h cycle of analyses of 2.2 ± 2.3 min (mean ± SD) and 95% limits of agreement from −2.3 to 6.7 min.

We then evaluated the effects of caffeine and doxepin using both sleep recording methods. Caffeine is an adenosine receptor antagonist that induces wakefulness^34^ and was administered at the beginning of the light period (i.e. rodent sleep time). As expected, caffeine reduced EEG/EMG-defined sleep time for 3 h after injection (Supplementary Fig. 8A). This result was confirmed by recording immobility from the digital video analysis (Supplementary Fig. 8B). The percentage of sleep measured by immobile episodes in both vehicle and caffeine groups (Supplementary Fig. 8C and D) was almost identical to EEG/EMG recording. For both vehicle and caffeine groups, immobility-defined and EEG/EMG-defined sleep had a correlation coefficient of 98% (Supplementary Fig. 8E and F). We observed in the vehicle group, a Bland–Altman estimated bias across the 24-h cycle of analyses of 3.4 ± 2.4 min (means ± SD) and 95% limits of agreement from −1.4 to 8.2 min (Supplementary Fig. 8G); and for the caffeine group, an estimated bias of 2.2 ± 2.2 min (means ± SD) with 95% limits of agreement between −2.2 and 6.6 min (Supplementary Fig. 8H).

Doxepin was administered at the start of the dark (i.e. mouse awake period) and increased sleep time for 2 h after injection as defined by the EEG/EMG recording (Supplementary Fig. 9A). Similar results were obtained by immobility-defined sleep analysis (Supplementary Fig. 9B). In both vehicle and doxepin groups (Supplementary Fig. 9C and D), immobility-defined sleep demonstrated a remarkable correlation with EEG/EMG-defined sleep. Immobility-defined sleep in response to vehicle and doxepin treatments (Supplementary Fig. 9E and F) showed a correlation coefficient of 99% and 94%, respectively, compared with EEG/EMG-defined sleep. For the vehicle group, we observed Bland–Altman estimated bias of 2.5 ± 2.0 min (means ± SD) and 95% limits of agreement from −1.4 to 6.5 min (Supplementary Fig. 9G) and, for the doxepin group, an estimated bias of 3.8 ± 2.8 min (means ± SD) with 95% limits of agreement from −1.6 to 9.2 min (Supplementary Fig. 9H).

### NTG-induced acute and chronic migraine-like pain does not affect immobility-defined sleep

**Figure 3:**
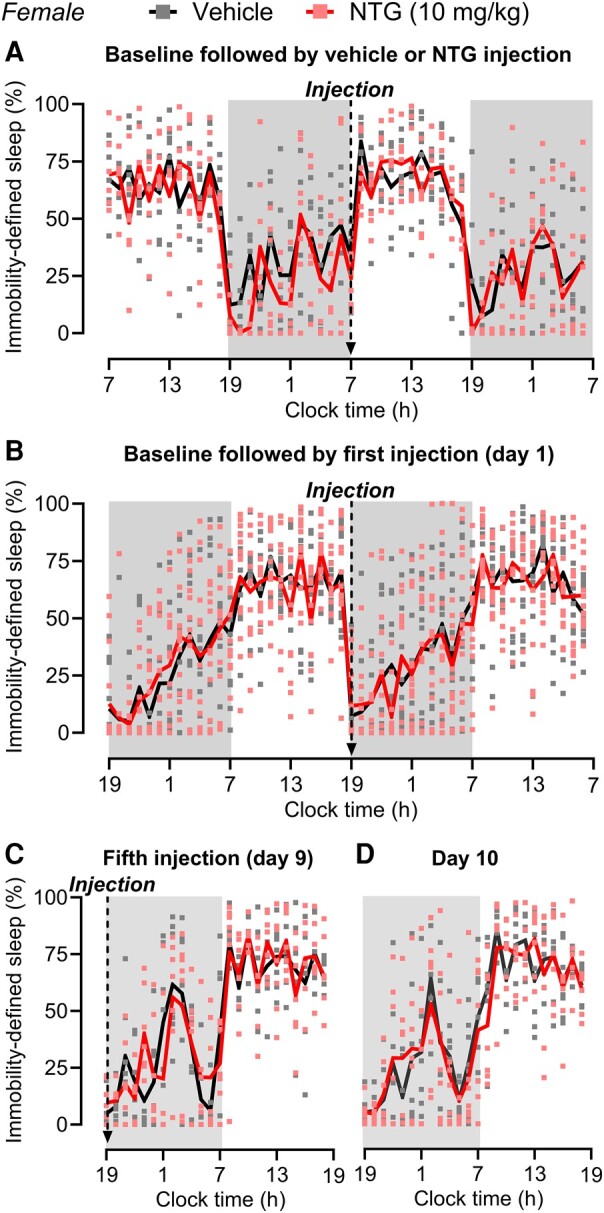
**NTG-induced acute and chronic migraine-like pain does not affect immobility-defined sleep in female mice. (A)** Following 24-h cycle baseline sleep recording using immobility measurements, female mice were treated with a single injection of NTG (10 mg/kg, i.p.) or vehicle at 7 a.m. (light phase onset), and immobility-defined sleep was recorded for 24-h cycle after injection. (**B–D**) In a separate group of female mice, baseline sleep was recorded for a 24-h cycle followed by an injection of NTG (10 mg/kg, i.p.) or vehicle every other day for 9 days (five injections in total) at 7 p.m. (dark phase onset). (**B**) The effect of NTG-induced acute migraine-like pain on sleep was evaluated after the first injection (Day 1). The effect of NTG-induced chronic migraine-like pain on sleep was evaluated after (**C**) the fifth injection (Day 9) and (**D**) the following day (Day 10). Total sleep time is expressed in the percentage of total time in 1-h bins over a 24-h cycle. Arrows indicate the times of injections. The dark phase is shaded in grey. Data were analysed by two-way repeated-measures ANOVA. (**A**) *F*(47, 564) = 1.212, *P* = 0.1632, *n* = 7 for both sham and NTG. (**B**) *F*(47, 1128) = 0.8658, *P* = 0.7271, *n* = 12 for sham, and *n* = 14 for NTG. (**C**) *F*(23, 276) = 1.011, *P* = 0.4517, *n* = 7 for both sham and NTG. (**D**) *F*(23, 276) = 0.5246, *P* = 0.9664, *n* = 7 for both vehicle and NTG. *F*- and *P*-values are shown for interaction factor (treatment and time). Data values for individual mice are shown as small symbols; lines represent the group means. NTG, nitroglycerin.

To eliminate any potential confounding factors of the implanted EEG/EMG head mount, we used immobility measurements to evaluate sleep in the following set of experiments. To evaluate the effect of acute migraine-like pain on sleep, female mice were treated with a single injection of NTG (10 mg/kg) at light phase onset after a 24-h baseline sleep recording. We did not observe a difference in the amount of immobility-defined sleep between NTG and vehicle groups during baseline recording and following a single NTG injection (Fig. 3A). The previous experiment with acute and chronic NTG treatment at dark phase onset was then replicated using only immobility measures. Female mice were randomly divided into vehicle and NTG treatment groups. Baseline immobility-defined sleep was recorded for a 24-h cycle, and no difference in the amount of sleep was observed between groups (Fig. 3B). Following acute systemic injection at the dark phase onset, NTG (10 mg/kg) did not change the amount of sleep as compared with vehicle (Fig. 3B). We then continuously treated the mice with NTG every other day for 9 days (five treatments in total) and recorded their sleep pattern. In agreement with our EEG/EMG sleep recordings, chronic migraine-like pain did not change the amount of immobility-defined sleep time on Day 9, i.e. recording performed immediately after the last NTG injection (Fig. 3C) or on the following day (Day 10) (Fig. 3D). Also, we did not observe alterations in immobility-defined sleep induced by NTG during the progression from acute to chronic migraine-like pain, i.e. from Days 2–8 (Supplementary Fig. 10A–G). Nine days after the last NTG or vehicle treatment, when the effects of NTG-induced pain were resolved, the female mice were treated with doxepin at the start of the dark period, as a positive control. As expected, doxepin promoted an increase in immobility-defined sleep as compared with the vehicle group, which lasted for about 4 h (Supplementary Fig. 10H).

### Dural IM-induced acute migraine-like pain does not affect immobility-defined sleep

**Figure 4:**
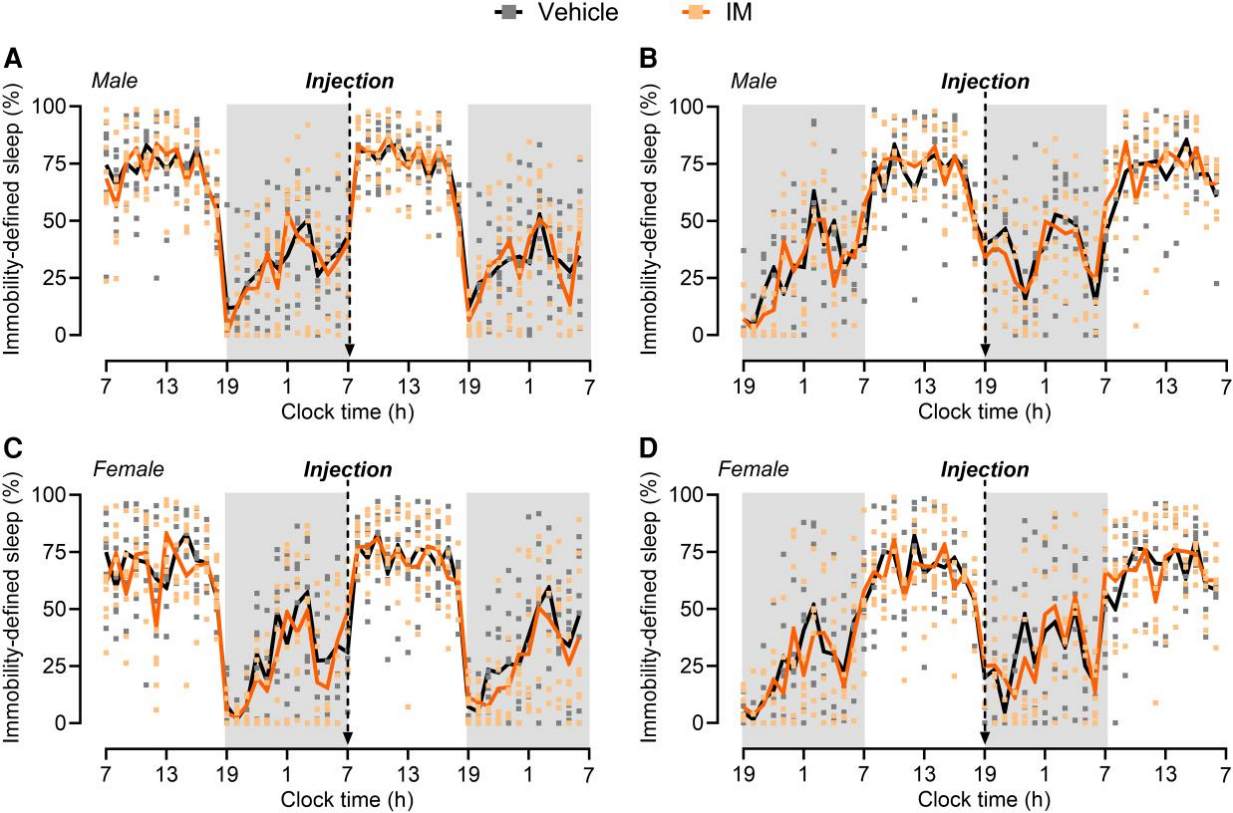
**Dural IM-induced acute migraine-like pain does not affect immobility-defined sleep in male or female mice.** Sleep time assessed by immobility measurements was recorded before (baseline) and immediately after a single dural injection of IM (5 uL) or vehicle (SIF) to induce acute migraine-like pain. In separate groups, male mice were treated either (**A**) at 7 a.m. (light onset) or (**B**) at 7 p.m. (dark phase onset). Similarly, female mice were treated either at (**C**) at 7 a.m. or (**D**) at 7 p.m. Total sleep time is expressed in the percentage of total time in 1-h bins over a 24-h cycle. Arrows indicate the times of injections. The dark phase is shaded in grey. Data were analysed by two-way repeated-measures ANOVA. (**A**) *F*(47, 658) = 0.6995, *P* = 0.9365, *n* = 8 for both sham and IM. (**B**) *F*(47, 517) = 1.006, *P* = 0.4650, *n* = 6 for vehicle, and *n* = 7 for IM. (**C**) *F*(47, 658) = 1.001, *P* = 0.4729, *n* = 8 for both sham and IM. (**D**) *F*(47, 517) = 0.6425, *P* = 0.9692, *n* = 6 for vehicle, and *n* = 7 for IM. *F*- and *P*-values are shown for interaction factor (treatment and time). Data values for individual mice are shown as small symbols; lines represent the group means. IM, inflammatory mediators.

We further evaluated the effect of acute migraine-like pain on sleep with dural IM injection followed by immobility assessment. Dural injections of IM have been consistently reported to induce migraine-like pain,^26^ a finding that was confirmed in the present experiments. Dural injection of IM induced both periorbital and hindpaw allodynia in both male (Supplementary Fig. 11A and B) and female (Supplementary Fig. 11C and D) mice. Baseline sleep was recorded for a 24-h cycle before IM injection, and no differences in the amount of sleep were observed between groups (Fig. 4A–D). In both male and female mice, dural IM injection either at the light phase onset (Fig. 4A and C) or immediately before the dark phase (Fig. 4B and D) did not affect the percentage of immobility-defined sleep as compared with the vehicle group.

### UMB-induced migraine-like pain does not affect immobility-defined sleep

**Figure 5:**
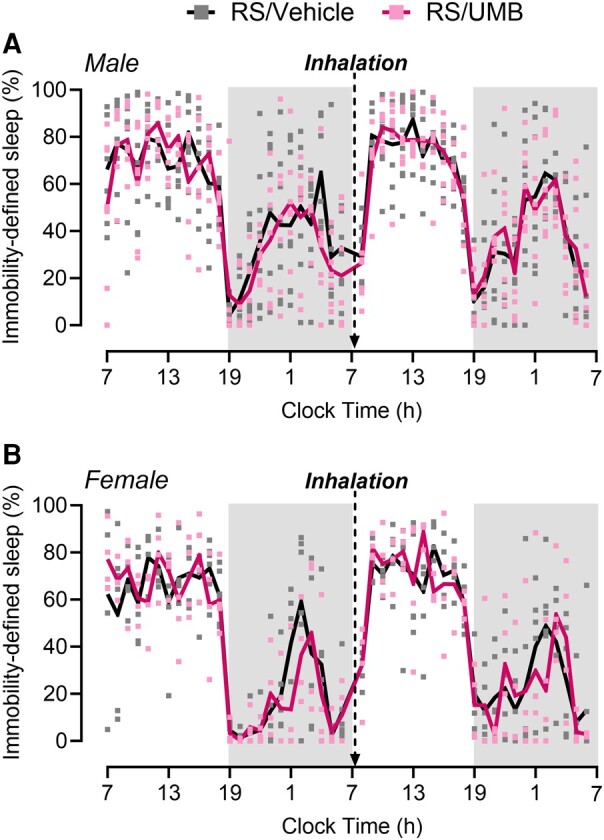
**UMB-induced migraine-like pain in RS-primed male and female mice does not affect immobility-defined sleep.** (**A**) Male and (**B**) female mice were subjected to RS for 2 h/day for 3 consecutive days. Three weeks later, sleep was recorded using immobility measurements 24 h before (baseline) and immediately after UMB (0.01 M/500 µL) or vehicle (phosphate-buffered saline) exposure by inhalation under isoflurane anaesthesia. Total sleep time is expressed in the percentage of total time in 1-h bins over a 24-h cycle. Arrows indicate the times of injections. The dark phase is shaded in grey. Data were analysed by two-way repeated-measures ANOVA. (**A**) *F*(46, 598) = 0.8020, *P* = 0.8224, *n* = 8 for RS/vehicle, and *n* = 7 for RS/UMB. (**B**) *F*(46, 368) = 0.9045, *P* = 0.6516, *n* = 6 for RS/vehicle, and *n* = 4 for RS/UMB. *F*- and *P*-values are shown for interaction factor (treatment and time). Data values for individual mice are shown as small symbols; lines represent the group means. RS, restraint stress; UMB, umbellulone.

Previously, we have characterized a novel injury-free preclinical model of migraine-like pain induced by UMB in RS-primed mice^25^ that was employed in the present studies (Supplementary Fig. 12A and B). To evaluate the effect of UMB-induced migraine-like pain in RS-primed mice on sleep, male and female RS-primed mice were exposed to UMB or vehicle by inhalation at 7:15 a.m. after a 24-h cycle baseline sleep recording. In both male (Fig. 5A) and female (Fig. 5B) RS-primed mice, UMB did not affect the amount of sleep predicted by immobility measurements as compared with vehicle.

### Subthreshold NTG and subthreshold CGRP induced migraine-like pain in sleep-deprived female mice

**Figure 6:**
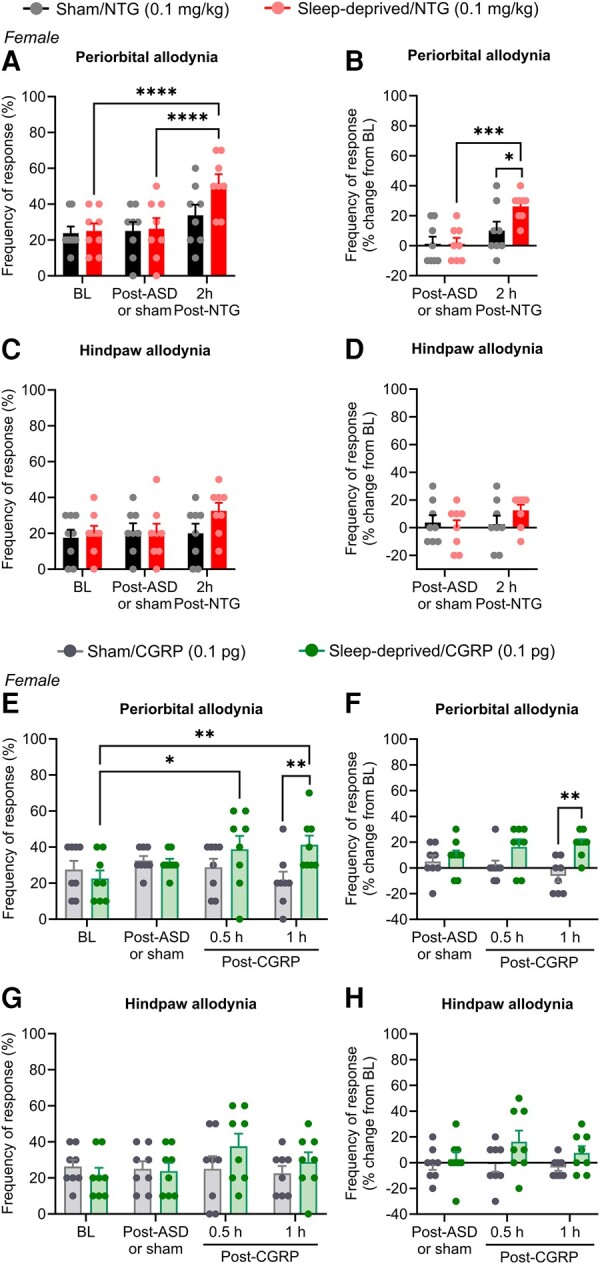
**Subthreshold dose of NTG or CGRP induces migraine-like pain in female sleep-deprived mice.** In female mice, **(A and B)** periorbital and (**C and D**) hindpaw frequency of response to tactile stimulation was assessed before (baseline) and immediately after 6-h acute sleep deprivation or sham condition. Then, both sleep-deprived and sham mice received a subthreshold dose of NTG (0.1 mg/kg, i.p.), and measurements were performed again 2 h after injection. In a separate group of female mice, **(E and F)** periorbital and (**G and H**) hindpaw tactile frequency of response was measured before and immediately after acute sleep deprivation or sham condition. Then, both sleep-deprived and sham mice received a dural injection of a subthreshold dose of CGRP (0.1 pg/5 μL), and measurements were performed again 0.5 and 1 h after injection. Cutaneous allodynia was evaluated using von Frey filaments (0.4 g to the periorbital and 0.6 g to the hindpaw region) and is expressed as a percentage of the frequency of response to tactile stimulation and its percentage change from baseline. Data were analysed by two-way repeated-measures ANOVA followed by Sidak’s or Tukey’s test. (**A**) *F*(2, 28) = 3.970, *P* = 0.0304. (**B**) *F*(1, 14) = 5.502, *P* = 0.0342. (**C**) *F*(2, 28) = 1.746, *P* = 0.1930. (**D**) *F*(1, 14) = 3.080, *P* = 0.1011. (**E**) *F*(3, 42) = 4.120, *P* = 0.0120. (**F**) *F*(1, 14) = 12.20, *P* = 0.0036, treatment factor. (**G**) *F*(3, 42) = 1.966, *P* = 0.1337. (**H**) *F*(2, 28) = 1.629, *P* = 0.2141. *F*- and *P*-values are shown for interaction factor (treatment and time), unless otherwise noted. *P < 0.05; **P < 0.01; ***P < 0.001; ****P < 0.0001. Data values for individual mice are shown as small symbols; bars represent the means ± SEM; *n* = 8 mice for all experimental groups. ASD, acute sleep deprivation; BL, baseline; NTG, nitroglycerin; CGRP, calcitonin gene-related peptide.

We employed a procedure of sleep deprivation induced by novel object placements^23^ that has not been associated with increased stress responses. Consistent with previous findings, sleep deprivation using this method did not alter corticosterone serum levels, suggesting a stress-free model of sleep deprivation (Supplementary Fig. 13A). A lack of stress was also supported by unaltered prolactin levels following acute sleep deprivation (Supplementary Fig. 13B). Using this method, we first evaluated the effect of a subthreshold dose of NTG on periorbital and hindpaw cutaneous allodynia immediately after sleep deprivation in female mice. The frequency of response for periorbital (Fig. 6A and B) and hindpaw (Fig. 6C and D) tactile cutaneous stimulation performed immediately after sleep deprivation was not changed from baseline and did not differ between sleep-deprived mice and sham mice (no sleep deprivation). In sham mice, NTG at 0.1 mg/kg did not modify periorbital (Fig. 6A and B) and hindpaw (Fig. 6C and D) frequency of response to tactile stimulation as compared with baseline and post-sleep deprivation measurements, thereby confirming that this was a subthreshold dose. However, in sleep-deprived mice, this subthreshold dose of NTG induced periorbital allodynia (Fig. 6A and B and Supplementary Fig. 14A) and produced a non-significant increase in hindpaw responses (Fig. 6C and D and Supplementary Fig. 14B) at 2 h after systemic injection of NTG. A separate cohort of female mice received a subthreshold dose of dural CGRP. Again, acute sleep deprivation did not induce either periorbital (Fig. 6E and F) or hindpaw (Fig. 6G and H) allodynia. In sham mice, dural injection of CGRP did not change the frequency of response to tactile stimulation, confirming that dural CGRP at 0.1 pg is a subthreshold dose in female mice. Consistent with the findings from the NTG experiment, in sleep-deprived mice, this subthreshold dose of dural CGRP induced periorbital allodynia (Fig. 6E and F) and produced a non-significant increased hindpaw response (Fig. 6G and H) at 0.5–1 h after injection.

**Figure 7:**
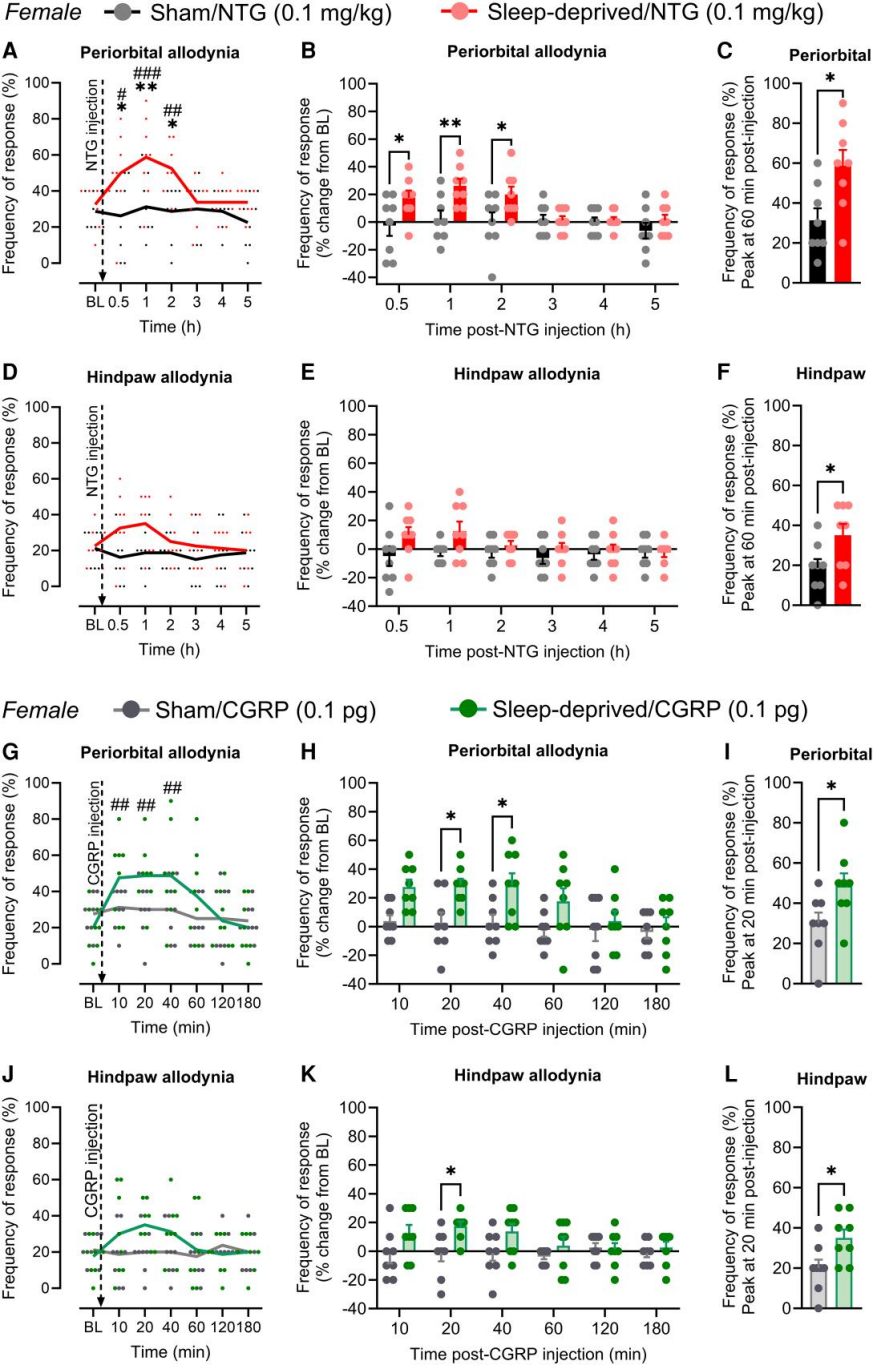
**Subthreshold dose of NTG or CGRP induces migraine-like pain on the following day of acute sleep deprivation in sleep-deprived female mice.** On the day following a 6-h acute sleep deprivation or sham conditions, (**A–C**) periorbital and (**D–F**) hindpaw frequency of response to tactile stimulation in female mice was measured before (baseline) and after systemic injection of a subthreshold dose of NTG (0.1 mg/kg, i.p.). In a separate group of female mice, (**G–I**) periorbital and (**J–L**) hindpaw frequency of response to tactile stimulation was measured before and after dural injection of a subthreshold dose of CGRP (0.1 pg/5 μL). Cutaneous allodynia was evaluated using von Frey filaments (0.4 g to the periorbital and 0.6 g to the hindpaw region) and is expressed as a percentage of the frequency of response to tactile stimulation and its percentage change from baseline. Data were analysed by (**A** and **B**, **D** and **E**, **G** and **H** and **J** and **K**) two-way repeated-measures ANOVA followed by Sidak’s or Tukey’s test or (**C**, **F**, **I**, **L**) Student’s *t*-test. (**A**) *F*(6, 84) = 3.609, *P* = 0.0031. (**B**) *F*(5, 70) = 3.316, *P* = 0.0096. (**C**) *t*(14) = 2.756, 0.0155. (**D**) *F*(6, 84) = 1.600, *P* = 0.1574. (**E**) *F*(5, 70) = 1.441, *P* = 0.2205. (**F**) *t*(14) = 2.263, *P* = 0.0401. (**G**) *F*(6, 84) = 2.807, *P* = 0.0154. (**H**) *F*(5, 70) = 5.331, *P* = 0.0003, treatment factor. (**I**) *t*(14) = 2.311, *P* = 0.0366. (**J**) *F*(6, 84) = 2.042, *P* = 0.0688. (**K**) *F*(1, 14) = 5.959, *P* = 0.0285, treatment factor. (**L**) *t*(14) = 2.510, *P* = 0.0250. *F*- and *P*-values are shown for interaction factor (treatment and time), unless otherwise noted. *P < 0.05; **P < 0.01; sham/NTG versus sleep-deprived/NTG or sham/CGRP versus sleep-deprived/CGRP. ^#^*P* < 0.05; ^##^*P* < 0.01; ^###^*P* < 0.001; sleep-deprived/NTG or sleep-deprived/CGRP post-injection versus baseline. Data values for individual mice are shown as small symbols; lines represent the group means; bars represent the means ± SEM; *n* = 8 mice for all experimental groups. BL, baseline; NTG, nitroglycerin; CGRP, calcitonin gene-related peptide.

On the day following sleep deprivation, the same animals received another subthreshold dose of systemic NTG or dural CGRP. In sleep-deprived mice, but not in mice without sleep deprivation, systemic subthreshold NTG induced robust periorbital allodynia consistent with migraine-like pain (Fig. 7A and B), with a peak effect at 60 min after injection (Fig. 7C). Significant hindpaw cutaneous allodynia (Fig. 7D–F) was also observed at 60 min after injection (Fig. 7F). Similarly, subthreshold dural CGRP promoted periorbital (Fig. 7G–I) and hindpaw (Fig. 7J–L) cutaneous allodynia only in sleep-deprived mice with the peak effect observed at 20 min after dural injection (Fig. 7I and L).

## Discussion

Migraine patients frequently report a range of sleep complaints,^2-10^ highlighting the presence of a complex and yet poorly understood relationship between migraine and sleep. To address this knowledge gap, we investigated a part of the migraine–sleep relationship with multiple preclinical models of migraine-like pain as well as in a stress-free model of sleep deprivation. Specifically, we explored whether the headache component of migraine can induce or disrupt sleep and whether sleep deprivation can serve as a priming mechanism to increase the likelihood of migraine attack by normally innocuous stimuli that can serve as triggers. We report that (i) acute and chronic migraine-like pain induced by systemic NTG in either the light (sleep period) or dark (active period) phase does not affect sleep quantity, depth or architecture of female mice; that (ii) acute migraine-like pain induced by dural IM either in the light or dark phase does not affect total sleep time of male and female mice; and that (iii) acute migraine-like pain induced by UMB, a TRPA1 agonist, in a priming model in the light phase did not affect total sleep time in male or female mice. Conversely, we show that a subthreshold dose of NTG or CGRP can induce migraine-like pain only in female mice with prior sleep deprivation.

Migraine exists on a spectrum, encompassing both episodic (less than 15 days with headache per month) and chronic (>15 days with headache per month) forms that vary in frequency and severity. Chronic migraine is characterized by headaches on 15 or more days per month for at least 3 months, with at least eight of those days per month fulfilling the criteria for migraine, while episodic migraine refers to a frequency of migraine and headache attacks that occur less frequently.^35^ As the majority of patients report the most likely onset of migraine in the morning,^31^ we administered systemic NTG to mice at the beginning of the dark (i.e. ‘morning’) phase to model both acute and chronic migraine-like pain. NTG is a nitric oxide donor that induces headaches in normal subjects and migraine headache in individuals with migraine.^36^ Preclinical studies show that NTG produces cutaneous allodynia consistent with migraine-like pain,^19-22^ which was confirmed in the present study. Nevertheless, NTG-induced acute and chronic migraine-like pain did not significantly alter multiple sleep measurements that were evaluated in our experiments.

The headache phase of migraine is thought to be caused by the activation and sensitization of meningeal nociceptors due to the release of neuroinflammatory and vasodilating peptides in the meninges that lead to trigeminovascular pathway activation.^37,38^ Thus, it seemed possible that the surgical implantation of the EEG/EMG head mounts could have influenced the outcomes of our studies. For this reason, we repeated the NTG experiment by using a non-invasive high-throughput method allowing evaluation of sleep duration in mice of both sexes.^29^ This method captures immobile episodes predicting the mouse sleep state.^29,39^ We first validated this approach by comparing immobility-defined sleep with EEG/EMG-defined sleep simultaneously in the same animals. We found a very high correlation of sleep state of 99% in baseline assessments of naïve mice. Similarly, a very high outcome correlation of the effects of caffeine, an adenosine receptor antagonist that induces wakefulness^34^ administered during the sleep phase, and doxepin, a first-generation H_1_R antagonist that induces sleep^24^ administered during the active phase, was observed with the two methods in the same animals. Corroborating outcomes from EEG/EMG recordings, NTG-induced acute and chronic migraine-like pain in female mice did not affect immobility-defined sleep. Similarly, direct activation of nociceptive trigeminal afferents with an acute dural injection of IM either in the sleep or active phases also did not affect sleep duration in either male or female mice assessed by immobility. These data suggest that the potential influence of the EEG/EMG electrode implantation had a minimal, if any effect, on sleep outcomes.

Provocation strategies for studying migraine, such as by administration of NTG to promote headache in healthy volunteers^40^ or headache-like pain in naïve animals,^19,20^ neglect the role of pre-existing vulnerability present in subjects with underlying migraine pathophysiology. Migraine has been characterized as a threshold disorder,^41^ where subthreshold stimuli could lead to headache attacks in vulnerable individuals due to changes in thresholds promoted by peripheral and/or central sensitization.^41^ Anecdotal reports have suggested that inhalation of UMB, a monoterpene ketone found in the California bay leaf *Umbellularia californica*, which activates the TRPA1 channel that detects environmental irritants,^42^ induces headache only in individuals with pre-existing headache disorders.^43^ We used a previously characterized novel injury-free preclinical model of migraine-like pain induced by UMB in primed mice.^25^ In this model, repeated RS induces latent sensitization, a primed state whereby a subthreshold stimulus, in this case, inhalation of UMB, promotes migraine-like pain.^25^ We have previously shown^25^ that UMB induces persistent periorbital allodynia consistent with migraine-like pain after its inhalation only in RS-primed mice. Here, we found that UMB did not change immobility-defined sleep time in RS-primed female and male mice.

Together, our data show that migraine-like pain induced either at the light or dark phase onset does not disrupt total sleep time and does not affect the sleep architecture of mice. Sex differences in sleep outcome measures were not observed. These findings suggest that subjective reports of decreased or increased sleep in migraine patients are unlikely to result directly from the pain component of the migraine headache. Common approaches for assessing sleep in patients rely on subjective outcomes from self-reported questionnaires, which have been shown to have a poor agreement with objective measures of sleep.^44^ Patients with migraine, as opposed to other headache types, overestimate their sleep latency as compared with objective measurements.^45^ Consequently, the perception of disrupted sleep resulting from migraine headaches could be inaccurate. A recent meta-analysis reported that, although migraine patients displayed a lower percentage of REM sleep and subjectively reported worse sleep quality, no robust evidence supported differences in several other objective sleep measures, including total sleep time, percentage of wake, NREM sleep and sleep efficiency in adult migraine patients compared with healthy controls,^6^ a conclusion that largely agrees with our preclinical findings. Patients often report increased sleep during a migraine attack. Migraine is characterized by heightened sensitivity to light (photophobia) and sounds (phonophobia),^46^ and during a migraine attack, patients often seek to be in dark and quiet environments. Under these circumstances, it seems reasonable to speculate that the greatly decreased sensory stimuli from these environments might contribute to migraine patients falling asleep during an attack. It should also be noted that patient perception of changes in sleep with migraine attacks could be related to the activation of the hypothalamus that occurs during the premonitory phase of migraine^47,48^ possibly altering sleep independently of pain. Changes in sleep microstructure in patients with migraine have been reported in the period preceding the pain phase.^17,49,50^

In the present study, we demonstrate that a single period of acute sleep deprivation did not induce allodynia, but acute sleep deprivation can act as a priming mechanism to induce a state of vulnerability to a normally subthreshold stimulus that may elicit migraine-like pain. A previous report showed that acute sleep deprivation could prolong hindpaw allodynia induced by NTG.^51^ We used a model of acute sleep deprivation induced by new object placement that did not affect circulating levels of corticosterone or prolactin, hormones associated with stress and migraine.^52-54^ As migraine is a female-prevalent disorder^55^ and the expression of allodynia and pericranial tenderness (signs of central sensitization) have been reported to be more prominent in women than men with primary headache,^56^ we focused our study on female mice. Following acute sleep deprivation, a normally subthreshold dose of either systemic NTG or supradural CGRP induced cutaneous allodynia in sleep-deprived mice, on both the day of sleep deprivation and the day following sleep deprivation. Cutaneous allodynia induced by subthreshold NTG and CGRP was more robust in the periorbital area than in the hindpaw, consistent with migraine-like pain, and more evident 24 h after sleep deprivation than on the same day of sleep deprivation. A previous human study^57^ reported no associations between sleep disruption and odds of migraine headache on the day immediately following the sleep period (Day 0) but found that low sleep efficiency (sleep fragmentation) increased the odds of migraine headache on the following day (Day 1), i.e. more than 24 h after sleep fragmentation, consistent with our findings. The precise mechanisms of how sleep deprivation increases the likelihood of migraine attack are unknown. It is possible to speculate that sleep disruption may promote decreased thresholds for activation of peripheral nociceptors as well as central sensitization so that sensory inputs are amplified, mechanisms that would be consistent with increased migraine attacks. Reduced sleep quantity, more than other sleep-related issues, has been associated with central sensitization in migraine patients, with a strong correlation with both allodynia and pericranial tenderness.^56^ Sleep deprivation has also been reported to increase cortical excitability in migraine patients,^58,59^ and preclinical studies have shown that sleep deprivation can enhance cortical spreading depolarization,^60^ suggesting the possibility that migraine aura could serve as a potential trigger for migraine attack in patients with disrupted sleep.^61^

Our data suggest that there is a directional causality in the relationship between migraine-like pain and sleep. While the pain phase of migraine does not disrupt sleep, sleep deprivation increases the likelihood of migraine-like pain. It should also be noted that increased sleep could increase the likelihood of migraine attack. Hypnic headache is a clinical syndrome that exclusively manifests during sleep^62^ raising the possibility that sleep may be permissive for migraine. Diminished neuronal activity in aminergic nuclei in the dorsal raphe and the locus coeruleus could decrease descending inhibition of afferent sensory traffic enhancing the likelihood of pain attacks.^63^ These possibilities will require future studies in preclinical models and in patients. Understanding and addressing the complex interplay between migraine and sleep may allow the implementation of therapeutic strategies including lifestyle modifications, behavioural interventions and pharmacological approaches to improve sleep quality for improved management of migraine.

## Supplementary Material

fcae051_Supplementary_Data

## Data Availability

All data are available upon reasonable request.
