# Supplementary material for: Unraveling the directional relationship of sleep and migraine-like pain
Source: Brain Commun. 2024 Feb 18;6(2):fcae051. doi: 10.1093/braincomms/fcae051 (PMC10914446; doi:10.1093/braincomms/fcae051)
Supplement: fcae051_Supplementary_Data [file fcae051_supplementary_data.docx]

**Experimental design**

**Effect of acute NTG, IM and UMB on tactile allodynia**

For both NTG and IM models, baseline periorbital and hindpaw frequency of response to tactile stimulation was measured in both female and male mice followed by single NTG (10 mg/kg, i.p.) or single dural IM injection. For the UMB model, male and female mice were subjected to repeated RS priming and, 16 days later, baseline periorbital frequency of response to tactile stimulation was measured followed by either UMB or vehicle (PBS) inhalation. Subsequent measurements of periorbital and hindpaw response frequencies to tactile stimulation were recorded at 30-minute intervals and then hourly following NTG, IM, or UMB treatment. For all the models baseline measurements and treatments were conducted in the morning, corresponding to the onset of the mouse sleep period.

**Effect of acute and chronic NTG-induced migraine-like pain on EEG/EMG- and immobility-defined sleep.**

EEG/EMG- and immobility-defined sleep were recorded in separate groups of female mice. After head-mount surgery recovery (EEG/EMG recording experiments) and acclimation to the experimental condition, baseline EEG/EMG- and immobility-defined sleep were recorded for a 24-h cycle (day 0) in all our experimental conditions. Mice were then treated with NTG (10 mg/kg, i.p.) every other day for 9 days (5 treatments in total) at 7 p.m (dark phase onset, i.e., mouse active period). This protocol has been previously reported to produce a progressive and sustained migraine-like pain lasting for 7 days beyond the last injection and allows the study of the progression of migraine from an episodic to a chronic state.^1,2^ To evaluate the effect of NTG-induced acute migraine-like pain on sleep, EEG/EMG was recorded immediately after the first injection. To evaluate the effect of NTG-induced chronic migraine-like pain, EGG/EMG was recorded again immediately after the last injection (day nine) and the following day (day 10). Immobility-defined sleep was recorded during the entire period of treatment (days one to nine) and the following day (day 10). Nine days after the last NTG injection, mice were treated with a single injection of doxepin at 7 p.m. or vehicle to induce sleep as a positive control, and EEG/EMG and immobility-defined sleep were recorded for a 24-h cycle post-treatment. In a distinct cohort, female mice were treated with a single injection of NTG (10 mg/kg, i.p.) at 7 a.m. and immobility-defined sleep was recorded for 24-h cycle post-treatment.

**Validation of immobility-defined sleep recording**

Male mice were subjected to the EEG/EMG head-mount implantation surgery and, after recovery, individually placed in the sleep chambers. After the acclimation period, EEG/EMG and video were simultaneously recorded in the same animals for a 24-h cycle before any treatment (naïve mice). Then, mice were treated with caffeine (20 mg/kg, i.p.), doxepin (15 mg/kg, i.p., antihistamine), or their respective vehicles (saline, 10 mL/kg), and EEG/EMG and video were recorded for another 24-h cycle. Caffeine, doxepin, and vehicle were administered to the same groups of mice in a counterbalanced fashion, beginning with caffeine/vehicle followed by doxepin/vehicle, with a 48–60 h interval given between the treatments. Caffeine or its vehicle was administrated at 7:30 a.m. to determine possible disruption of sleep while doxepin or its vehicle was given at 6:45 p.m to promote sleep.

**Effect of acute IM-induced migraine-like pain on immobility-defined sleep**

Male and female mice were acclimated to the experimental conditions and baseline immobility-defined sleep was recorded for a 24-h cycle. Then, mice were treated with a single dural injection of IM mixture or vehicle (SIF) either at 7 a.m. or 7 p.m. Immobility-defined sleep was recorded again for 24-h post-treatment.

**Effect of UMB-induced migraine-like pain in RS-primed mice on immobility-defined sleep.**

Male and female mice were subjected to repeated RS priming and, 3 weeks later, baseline immobility-defined sleep was recorded for a 24-h cycle. Then, mice were treated with UMB or vehicle (PBS) at 7:15 a.m., and immobility-defined sleep was recorded again for another 24-h cycle post-treatment.

**Effect of acute subthreshold dose of NTG or CGRP inducing allodynia in sleep-deprived mice**

Periorbital and hindpaw allodynia were measured before and immediately after acute sleep deprivation or sham condition in female mice. Then, sleep-deprived and sham mice were both treated with a systemic subthreshold dose of NTG (0.1 mg/kg, i.p.) or dural CGRP (0.1 pg/ 5 μL), and tactile frequency of response was measured at 1-3 h (NTG group) or 0.5 and 1 h (CGRP group) post-injection. On the day following acute sleep deprivation, periorbital and hindpaw tactile frequency of response were assessed before and after a systemic subthreshold dose of NTG (0.1 mg/kg, i.p.) or dural CGRP (0.1 pg/ 5μL).

**
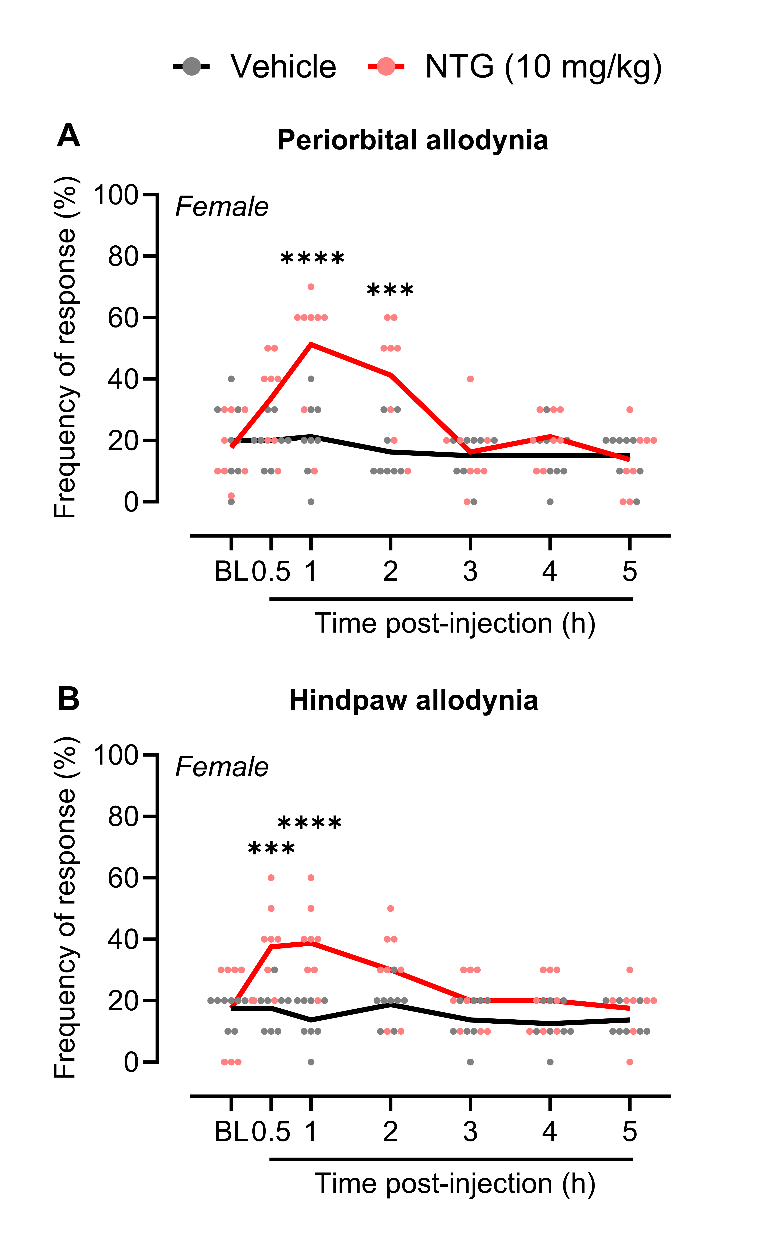
**

**Supplementary Figure 1. NTG induces robust periorbital and hindpaw allodynia consistent with migraine-like pain in female mice.** Female mice received a single injection of NTG (10 mg/kg, i.p.) or vehicle in the morning, and (**A**) periorbital and (**B**) hindpaw frequency of response to tactile stimulation were measured over 5-h post-treatment. Data were analyzed by two-way repeated-measures ANOVA followed by Sidak’s multiple comparisons. (**A**) F(6, 84) = 8.386, *P* < 0.0001. (**B**) F(6, 84) = 7.346, *P* < 0.0001. F- and *P*-values are shown for interaction factor (treatment and time). ****P* < 0.001; *****P* < 0.0001 NTG vs. vehicle. Data values for individual mice are shown as small symbols; lines represent the group means; n = 8 mice for all experimental groups. BL, baseline; NTG, nitroglycerin.


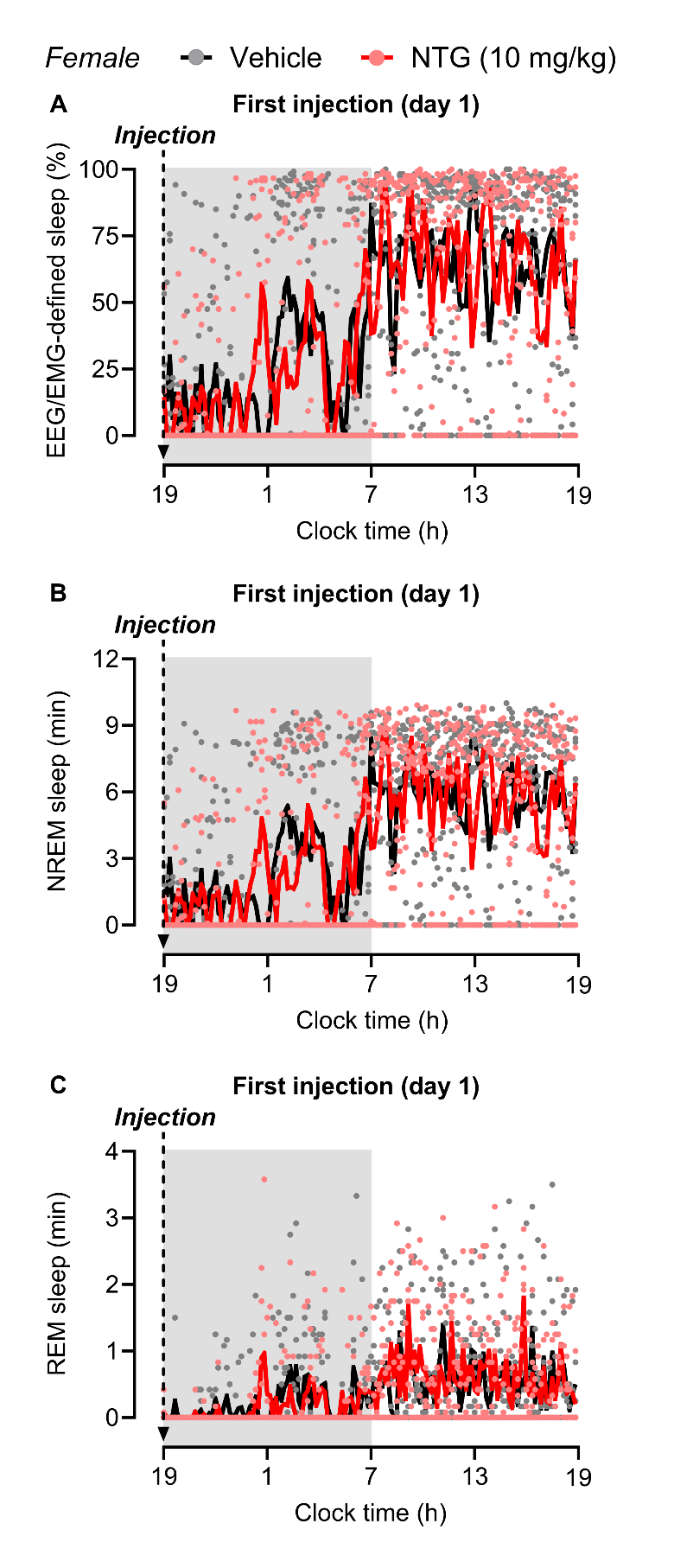


**Supplementary Figure 2. NTG-induced acute migraine-like pain does not affect the sleep quantity of female mice**. Female mice were implanted with an EEG/EMG head-mount to record multiple sleep measures. (**A**) Total sleep, (**B**) NREM, and (**C**) REM sleep time were recorded for a 24-h cycle immediately after a single systemic injection of NTG (10 mg/kg, i.p.) or vehicle at 7 p.m. to induce acute migraine-like pain. Total sleep, NREM, and REM sleep are expressed in 10-min bins. Arrows indicate the times of injections. The dark phase is shaded in grey. Data were analyzed by two-way repeated-measures ANOVA. (**A**) F(143, 1287) = 0.8994, *P* = 0.7894. (**B**) F(143, 1287) = 0.8862, *P* = 0.8211. (**C**) F(143, 1287) = 0.9414, *P* = 0.6725. F- and *P*-values are shown for interaction factor (treatment and time). Data values for individual mice are shown as small symbols; lines represent the group means; n = 6 for vehicle, and n = 5 for NTG. EEG, electroencephalogram; EMG, electromyography; NREM, non-rapid eye movement; NTG, nitroglycerin; REM, rapid-eye movement.


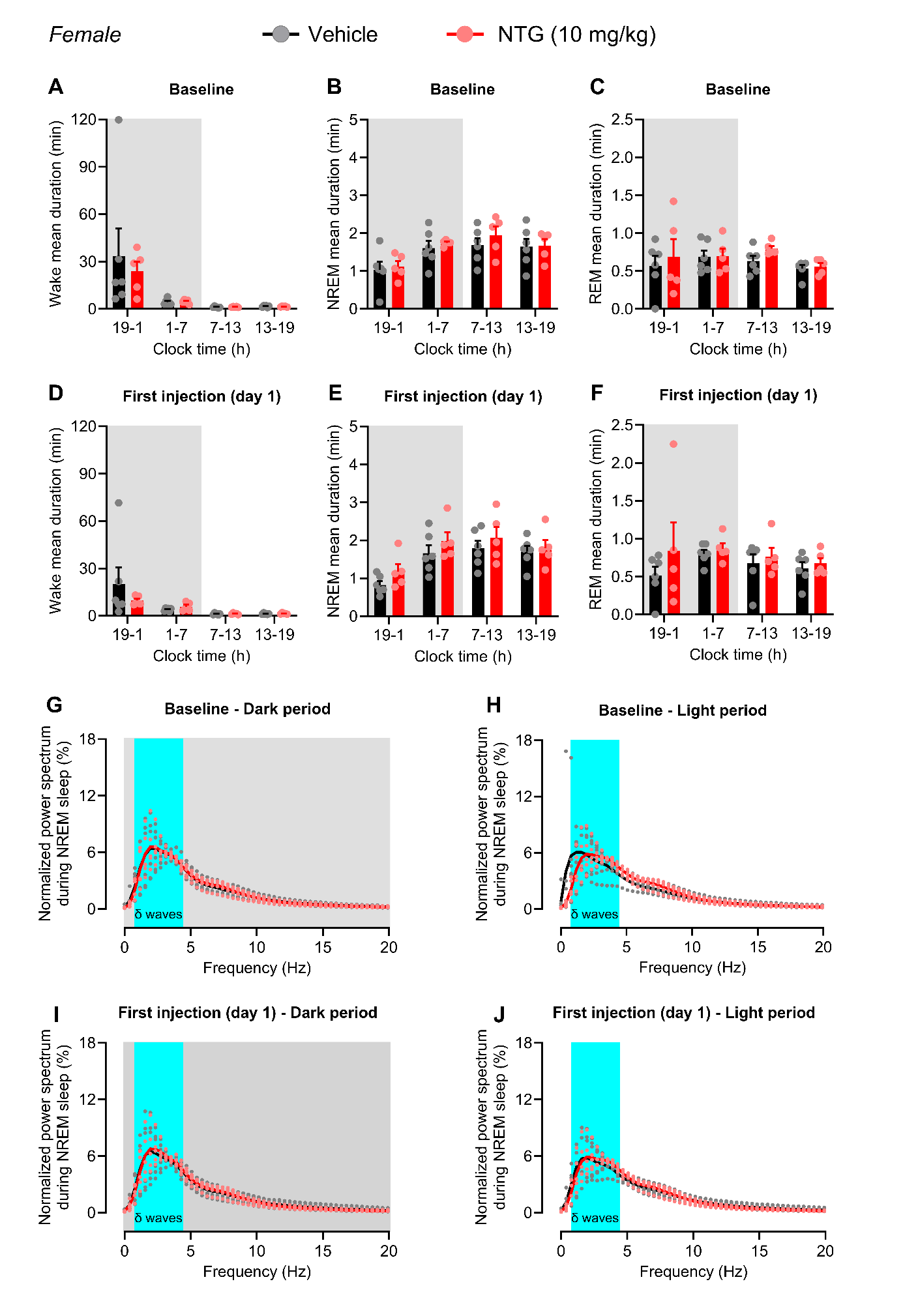


**Supplementary Figure 3. NTG-induced acute migraine-like pain does not affect the sleep architecture of female mice.** Female mice were implanted with an EEG/EMG head-mount to record multiple sleep measures before (baseline) and immediately after a single systemic injection of NTG (10 mg/kg, i.p.) or vehicle at 7 p.m. The mean durations of wake, NREM, and REM episodes are expressed in 6-h bins (early/late light and dark phases) over a 24-h cycle and were recorded at (**A-C**) baseline and (**D-F**) immediately after the first injection. NREM normalized power spectra during (**G**) the dark and (**H**) light phase at baseline, and during (**I**) the dark and (**J**) light phase following the first NTG injection. Delta (δ) wave (0.65-4.5Hz) is shaded in blue. The dark phase is shaded in grey. Data were analyzed by two-way repeated-measures ANOVA. (**A**) F(3, 27) = 0.2256, *P* = 0.8778. (**B**) F(3, 27) = 0.5045, *P* = 0.6824. (**C**) F(3, 27) = 0.3920, *P* = 0.7597. (**D**) F(3, 27) = 0.9094, *P* = 0.4495. (**E**) F(3, 27) = 0.3438, *P* = 0.7939. (**F**) F(3, 27) = 0.3899, *P* = 0.7612. (**G**) F(51, 459) = 0.0576, *P* > 0.9999. (**H**) F(51, 459) = 1.364, *P* = 0.0543. (**I**) F(51, 459) = 0.1138, *P* > 0.9999. (**J**) F(51, 459) = 0.3482, *P* > 0.9999. F- and *P*-values are shown for interaction factor (treatment and time). Data values for individual mice are shown as small symbols; lines represent the group means; bars represent the means ± SEM; n = 6 mice for vehicle, and n = 5 mice for NTG. NREM, non-rapid eye movement; NTG, nitroglycerin; REM, rapid-eye movement.


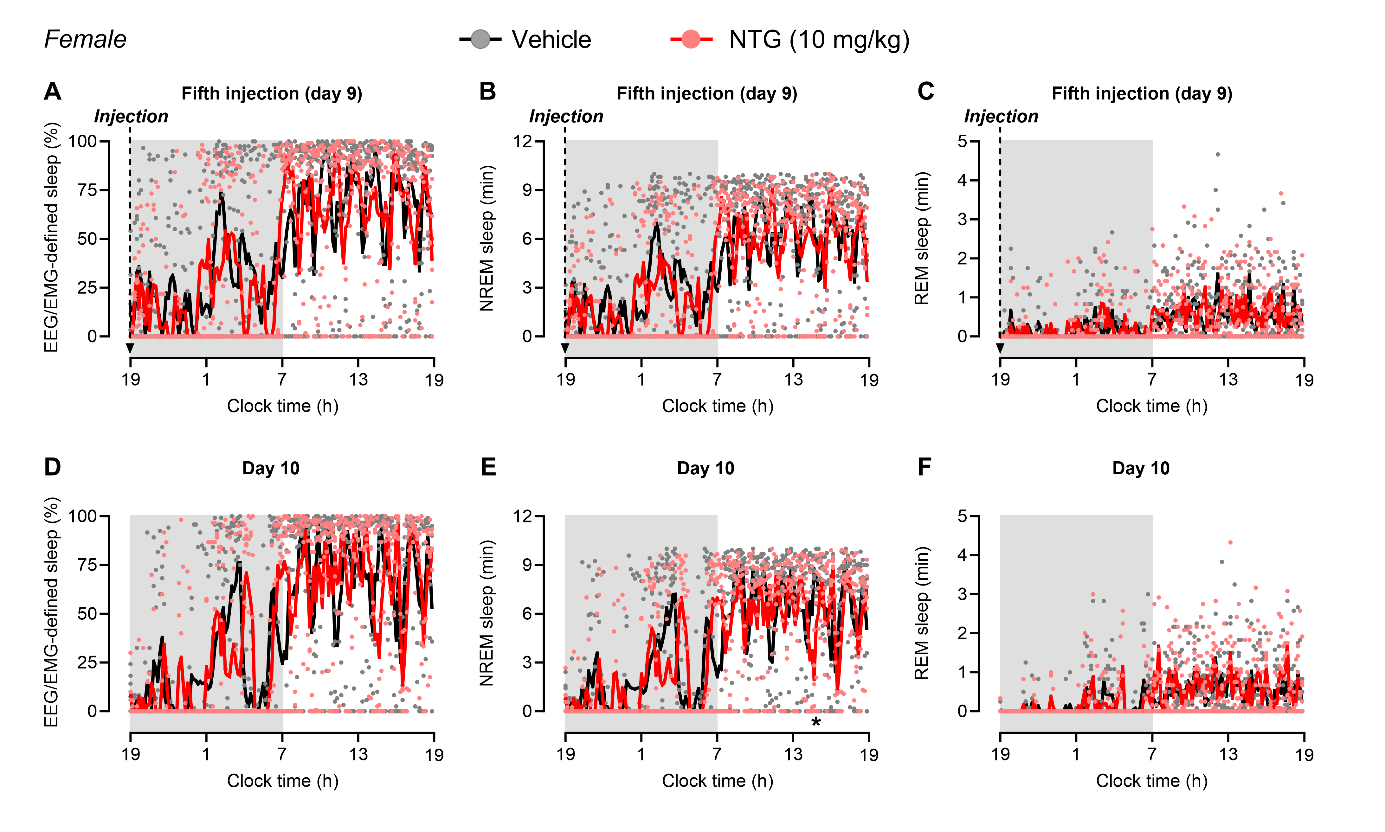


**Supplementary Figure 4. NTG-induced chronic migraine-like pain does not affect the sleep quantity of female mice**. Female mice were treated with NTG (10 mg/kg, i.p.) or vehicle every other day for 9 days (5 injections in total) at 7 p.m. to model a chronic migraine-like pain. Total sleep, NREM, and REM sleep time were measured (**A-C**) immediately after the fifth injection (day nine) and (**D-F**) the following day (day 10) using EEG/EMG recordings. Total sleep, NREM, and REM sleep are expressed in 10-min bins. Arrows indicate the times of injections. The dark phase is shaded in grey. Data were analyzed by two-way repeated-measures ANOVA. (**A**) F(143, 1287) = 0.8729, *P* = 0.8501. (**B**) F(143, 1287) = 0.8498, *P* = 0.8932. (**C**) F(143, 1287) = 1.009, *P* = 0.4571. (**D**) F(143, 1287) = 1.408, *P* = 0.0018. (**E**) F(143, 1287) = 1.438, *P* = 0.0010. (**F**) F(143, 1287) = 0.9226, *P* = 0.7277. F- and *P*-values are shown for interaction factor (treatment and time). Data values for individual mice are shown as small symbols; lines represent the group means; n = 6 for vehicle, and n = 5 for NTG. EEG, electroencephalogram; EMG, electromyography; NREM, non-rapid eye movement; NTG, nitroglycerin; REM, rapid-eye movement.


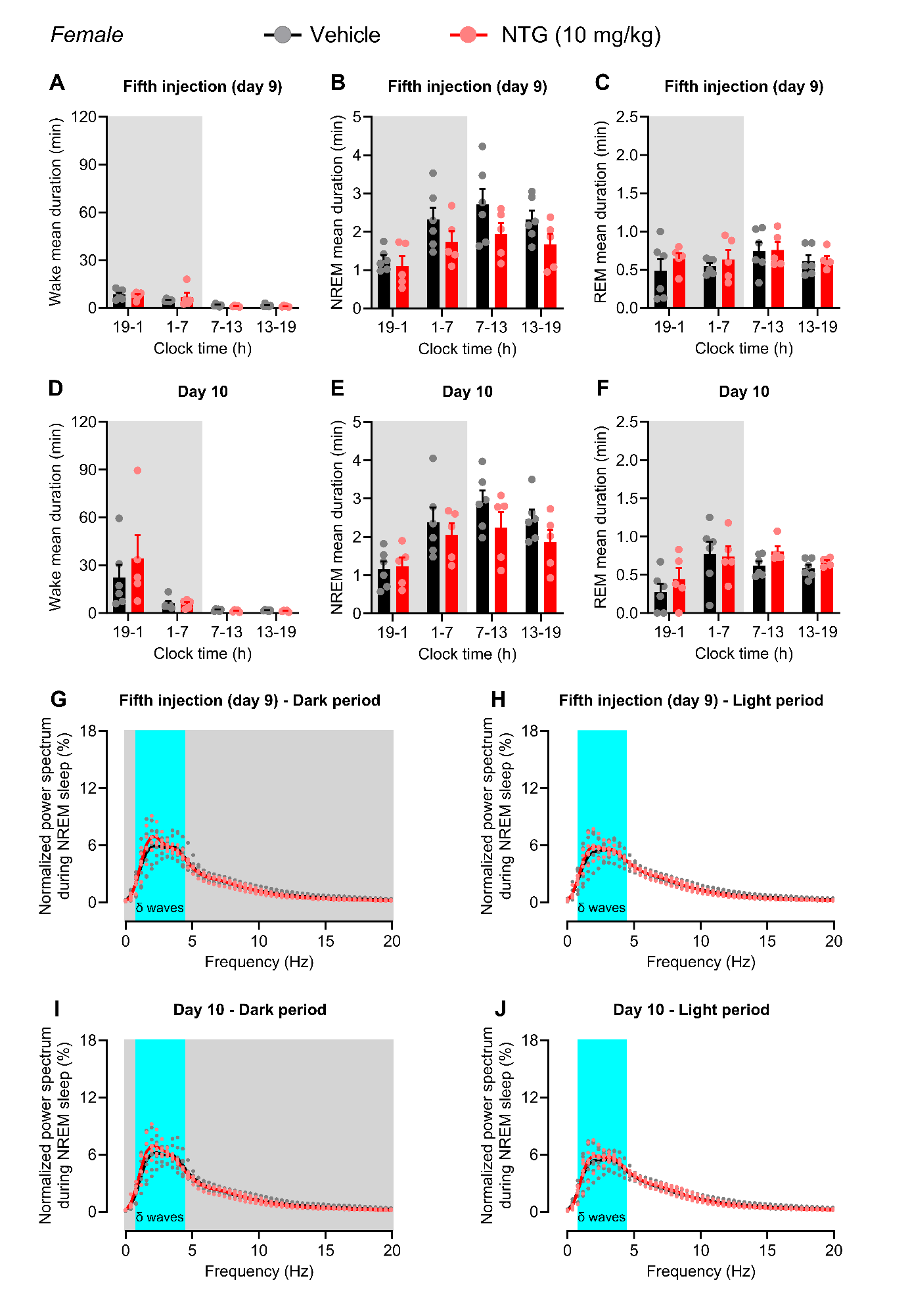


**Supplementary Figure 5. NTG-induced chronic migraine-like pain does not affect the sleep architecture of female mice.** Female mice were treated with NTG (10 mg/kg, i.p.) or vehicle every other day for 9 days (5 injections in total) at 7 p.m. to model a chronic migraine-like pain. The mean durations of wake, NREM, and REM episodes are expressed in 6-h bins (early/late light and dark phases) over a 24-h cycle and were measured (**A-C**) immediately after the fifth injection (day nine) and (**D-F**) the following day (day 10) using EEG/EMG recordings. NREM normalized power spectra during (**G**) the dark and (**H**) light phase on day nine and (**I**) the dark and (**J**) light phase on day 10. Delta (δ) wave (0.65-4.5 Hz) is shaded in blue. The dark phase is shaded in grey. Data were analyzed by two-way repeated-measures ANOVA. (**A**) F(3, 27) = 0.7048, *P* = 0.5575. (**B**) F(3, 27) = 1.162, *P* = 0.3424. (**C**) F(3, 27) = 0.4210, *P* = 0.7394. (**D**) F(3, 27) = 0.5909, *P* = 0.6263. (**E**) F(3, 27) = 1.767, *P* = 0.1773. (**F**) F(3, 27) = 0.6446, *P* = 0.5931. (**G**) F(51, 459) = 0.5000, *P* = 0.9985. (**H**) F(51, 459) = 0.3028, *P* > 0.9999. (**I**) F(51, 459) = 0.6900, *P* = 0.9488. (**J**) F(51, 459) = 0.3015, *P* > 0.9999. F- and *P*-values are shown for interaction factor (treatment and time). Data values for individual mice are shown as small symbols; lines represent the group means; bars represent the means ± SEM; n = 6 mice for vehicle, and n = 5 mice for NTG. NREM, non-rapid eye movement; NTG, nitroglycerin; REM rapid-eye movement.

***
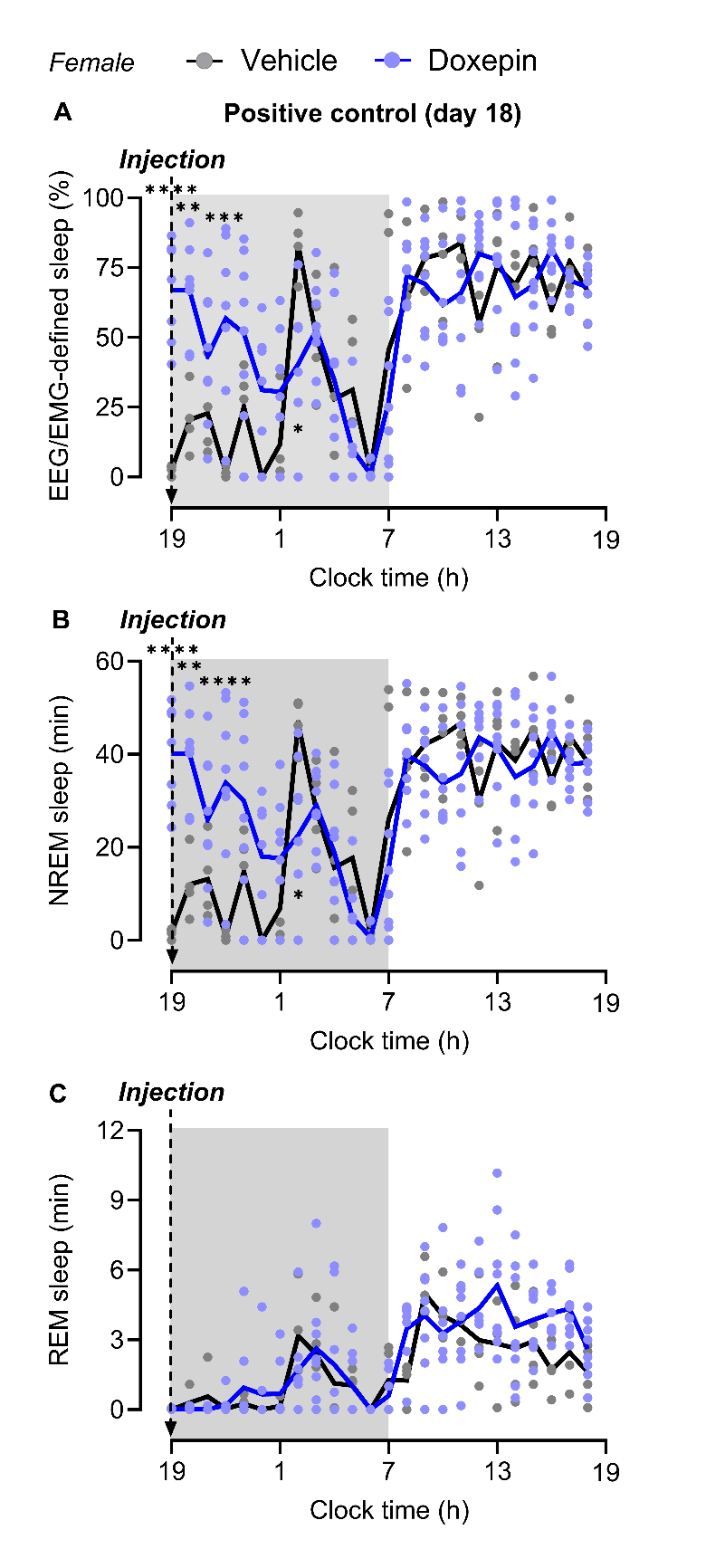
***

**Supplementary Figure 6. Doxepin increases NREM sleep in female mice.** As a positive control for EEG/EMG recordings and analysis, female mice were treated with doxepin (15 mg/kg, i.p.) or vehicle at 7 p.m. (**A**) total sleep time expressed in percentage of total time in 1-h bins over a 24-h cycle, (**B**) NREM sleep, and (**C**) REM sleep times were evaluated. Arrows indicate the time of the injections. The dark phase is shaded in grey. Data were analyzed by two-way repeated-measures ANOVA followed by Sidak’s multiple comparison test when appropriate. (**A**) F(23, 230) = 4.146, *P* < 0.0001. (**B**) F(23, 230) = 4.723, *P* < 0.0001. (**C**) F(23, 230) = 1.282, *P* = 0.1808. F- and *P*-values are shown for interaction factor (treatment and time). *P < 0.05; ***P* < 0.01; ****P* < 0.001 *****P* < 0.0001 doxepin vs. vehicle. Data values for individual mice are shown as small symbols; lines represent the group means; n = 4 mice for vehicle and n = 8 mice for doxepin. EEG, electroencephalogram; EMG, electromyography; NREM, non-rapid eye movement; REM rapid-eye movement.

**
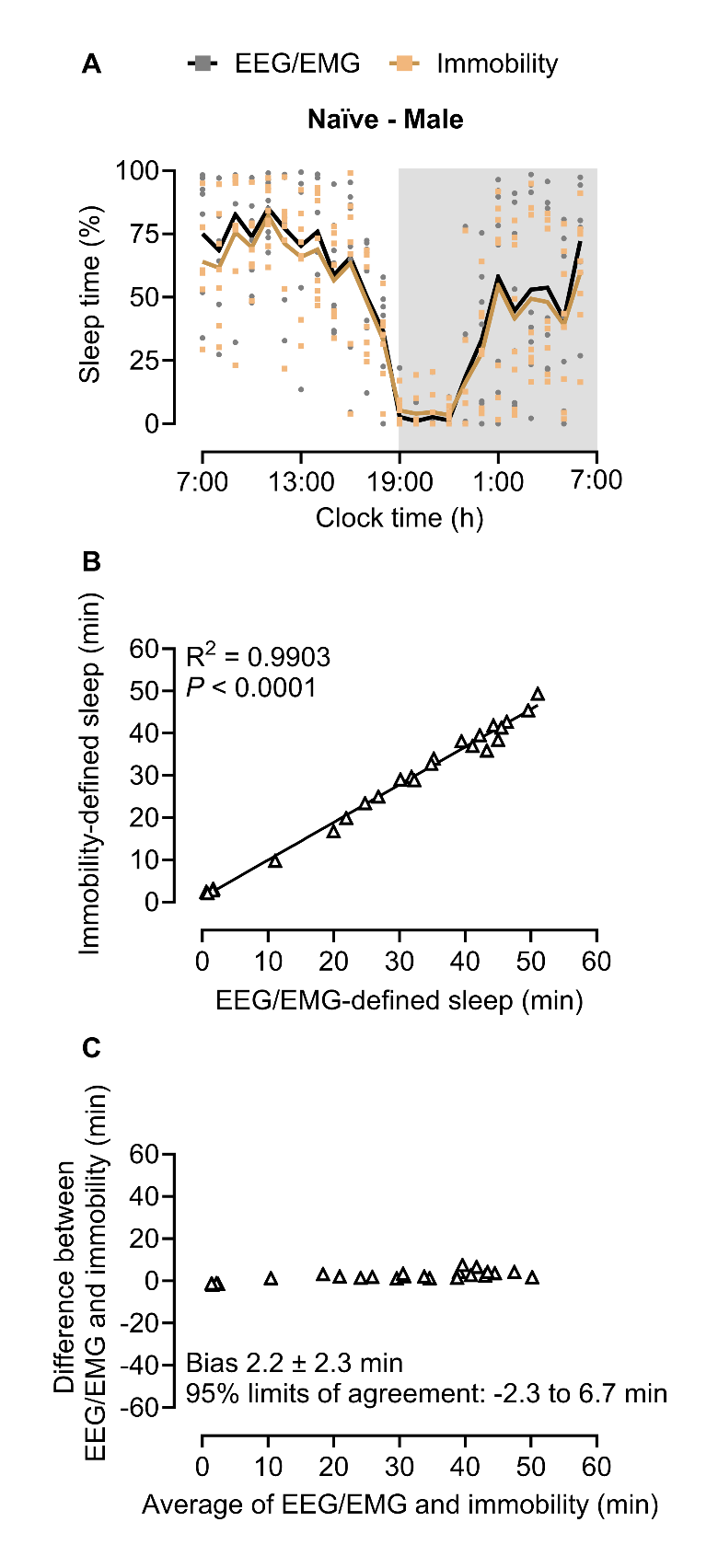
**

**Supplementary Figure 7. Immobile measurements correlate with EEG/EMG-defined sleep of naïve mice.** EEG/EMG and video were simultaneously recorded in male naïve mice. Video recordings were retrospectively analyzed by ANY-maze to evaluate mouse immobility as a noninvasive method to measure sleep. (**A**) Comparison between 24-h cycle EEG/EMG- and immobility-defined sleep recordings. Total sleep time is expressed as the percentage of total time in 1-h bins over a 24-h cycle. The dark phase is shaded in grey. Data were analyzed by two-way repeated-measures ANOVA. F(23, 322) = 0.1075, *P* > 0.9999, interaction factor (treatment and time). Data values for individual mice are shown as small symbols; lines represent the group means; n = 8 mice for both EEG/EMG and immobility recordings. (**B**) Pearson’s correlation was performed to evaluate the linear correlation between EEG/EMG- and immobility-defined sleep in 1-h bins over a 24-h cycle. r = 0.9951, r^2^ = 0.9903, *P* < 0.0001. (**C**) Bland-Altman analysis was performed to evaluate the agreement between immobility- and EEG/EMG-defined sleep in 1-h bins over a 24-h cycle. Bias 2.2 ± 2.3 min (mean ± SD) and 95% limits of agreement from -2.3 to 6.7 min. EEG, electroencephalogram; EMG, electromyography.

**
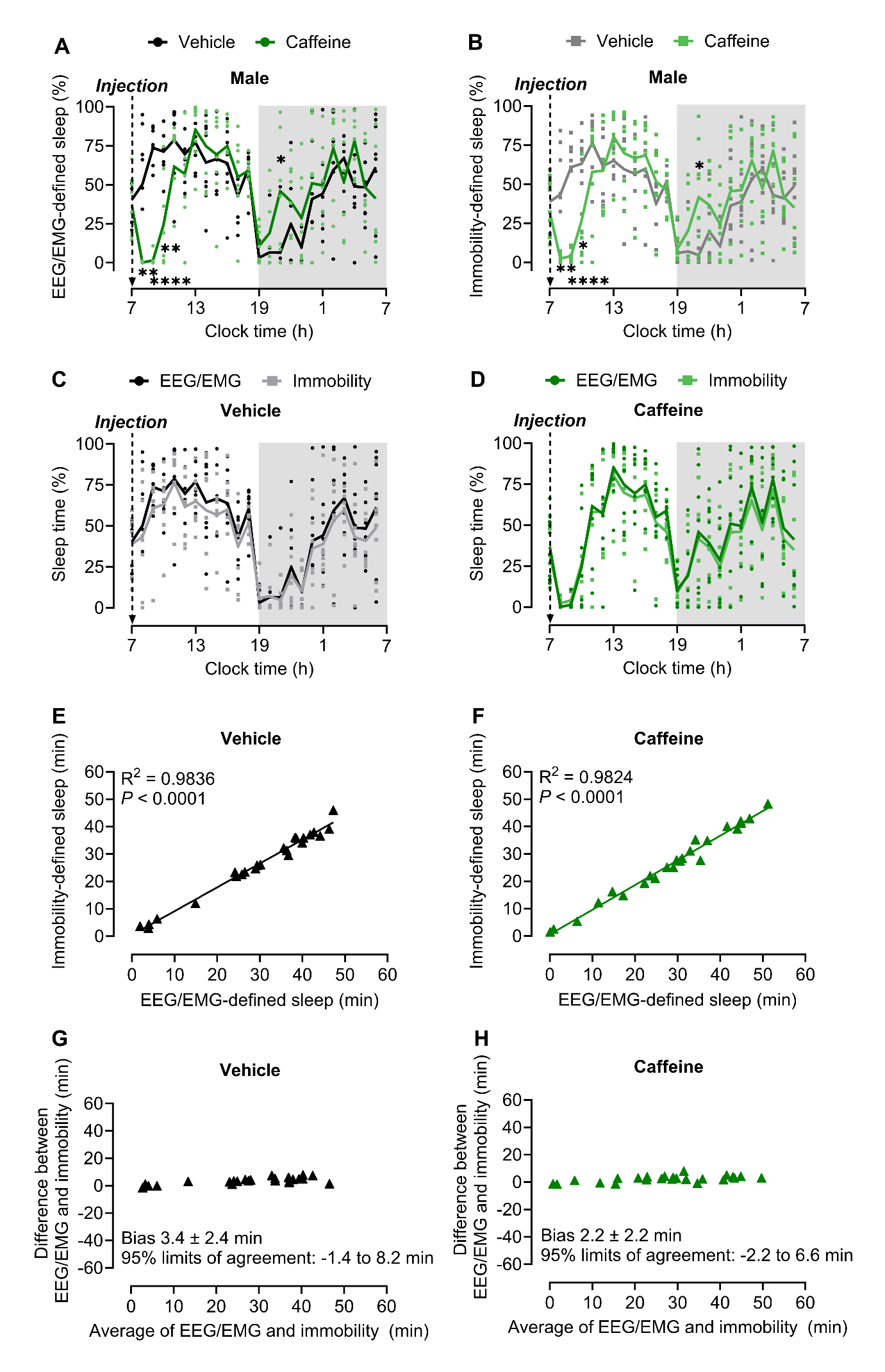
Supplementary Figure 8. Immobility measurements correlate with EEG/EMG-defined sleep in response to caffeine.** EEG/EMG and video were simultaneously recorded in male mice after a single injection of caffeine (20 mg/kg, i.p.) or vehicle at 7:30 a.m. Video recordings were posteriorly analyzed by ANY-maze to evaluate the effect of caffeine on immobility as a noninvasive method to predict sleep. (**A**) Effect of caffeine on EEG/EMG-defined sleep. (**B**) Effect of caffeine on immobility-defined sleep. Comparison between EEG/EMG- and immobility-defined sleep in response to treatment with (**C**) vehicle and (**D**) caffeine. Total sleep time is expressed in the percentage of total time in 1-h bins over a 24-h cycle. Arrows indicate the time of the injections. The dark phase is shaded in grey. (**A**-**D**) Data were analyzed by two-way repeated-measures ANOVA followed by Sidak’s multiple comparison test when appropriate. (**A**) F(23, 322) = 4.166, *P* < 0.0001. (**B**) F(23, 322) = 3.823, *P* < 0.0001. (**C**) F(23, 322) = 0.1347, *P* > 0.9999. (**D**) F(23, 322) = 0.08819, *P* > 0.9999. F- and *P*-values are shown for interaction factor (treatment and time). **P* < 0.05; ***P* < 0.01; *****P* < 0.0001; caffeine vs. vehicle. Data values for individual mice are shown as small symbols; lines represent the group means; n = 8 mice for both vehicle and caffeine. Pearson’s correlations were performed to evaluate the linear correlation between EEG/EMG- and immobility-defined sleep in response to treatment with (**E**) vehicle and (**F**) caffeine in 1-h bins over a 24-h cycle. (**E**) r = 0.9918, r^2^ = 0.9836, *P* < 0.0001. (**F**) r = 0.9912, r^2^ = 0.9824, *P* < 0.0001. Bland-Altman analyses were performed to evaluate the agreement between immobility- and EEG/EMG-defined sleep in response to treatment with (**G**) vehicle or (**H**) caffeine in 1-h bins over a 24-h cycle. (**G**) Bias 3.4 ± 2.4 min (means ± SD) and 95% limits of agreement from -1.4 to 8.2 min. (**H**) Bias 2.2 ± 2.2 min (means ± SD) with 95% limits of agreement between -2.2 and 6.6 min. EEG, electroencephalogram; EMG, electromyography.

**
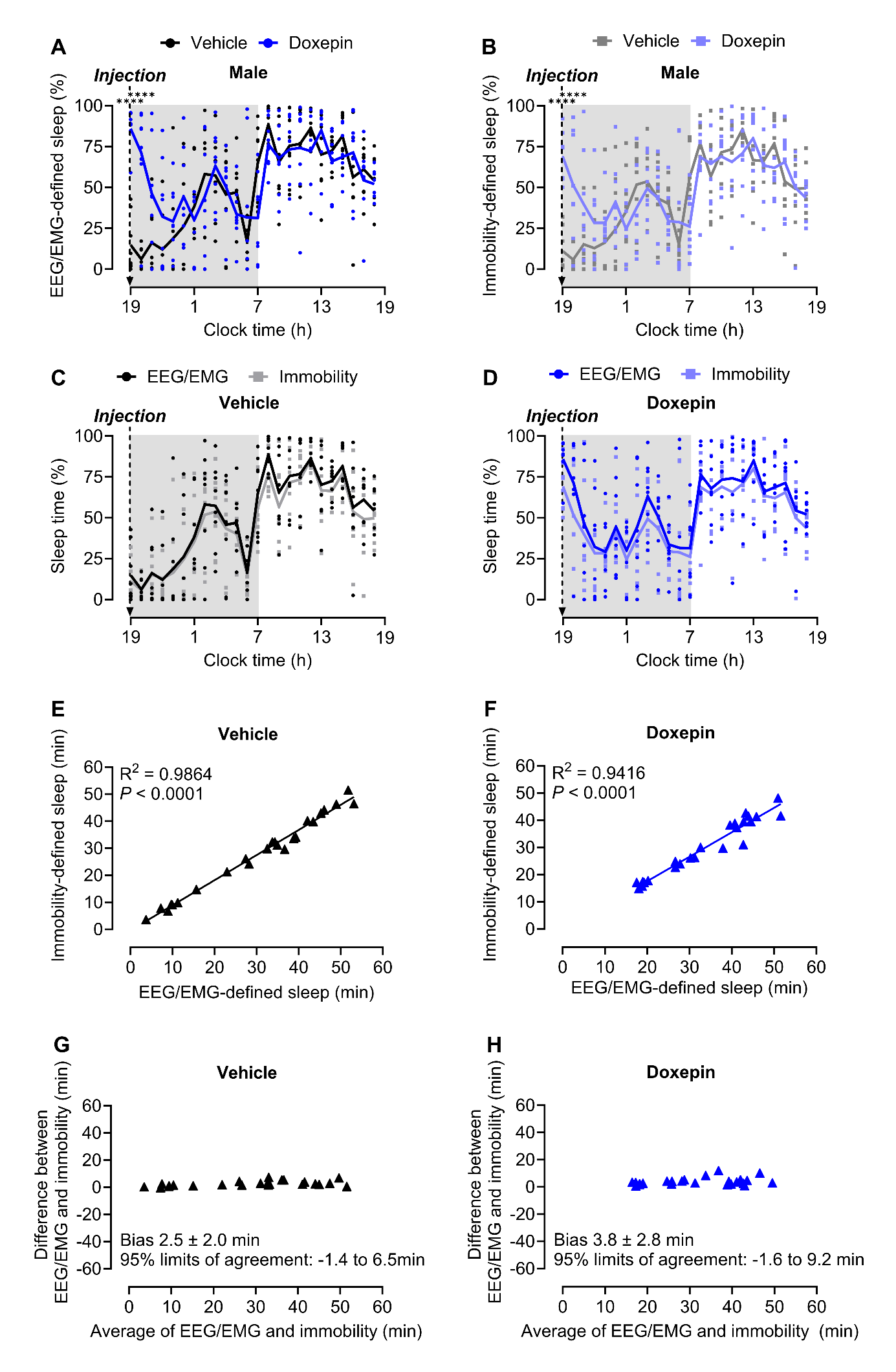
Supplementary Figure 9. Immobile measurements correlate with EEG/EMG-defined sleep in response to doxepin.** EEG/EMG and video were simultaneously recorded in male mice after a single injection of doxepin (15 mg/kg, i.p.) or vehicle at 7 p.m. Video recordings were retrospectively analyzed by ANY-maze to evaluate the effect of doxepin on immobility as a noninvasive method to measure sleep. (**A**) Effect of doxepin on EEG/EMG-defined sleep. (**B**) Effect of doxepin on immobility-defined sleep. Comparison between EEG/EMG- and immobility-defined sleep in response to treatment with (**C**) vehicle and (**D**) doxepin. Total sleep time is expressed as the percentage of total time in 1-h bins over a 24-h cycle. Arrows indicate the time of the injections. The dark phase is shaded in grey. (**A**-**D**) Data were analyzed by two-way repeated-measures ANOVA followed by Sidak’s multiple comparison test when appropriate. (**A**) F(23, 322) = 4.216, *P* < 0.0001. (**B**) F(23, 322) = 3.614, *P* < 0.0001. (**C**) F(23, 322) = 0.1069, *P* > 0.9999. (**D**) F(23, 322) = 0.1555, *P* > 0.9999. F- and *P*-values are shown for interaction factor (treatment and time). *****P* < 0.0001 doxepin vs. vehicle. Data values for individual mice are shown as small symbols; lines represent the group means; n = 8 mice for both vehicle and doxepin. Pearson’s correlations were performed to evaluate the linear correlation between EEG/EMG- and immobility-defined sleep in response to treatment with (**E**) vehicle and (**F**) doxepin in 1-h bins over a 24-h cycle. (**E**) r = 0.9932, r^2^ = 9864, *P* < 0.0001. (**F**) r = 0.9704, r^2^ = 0.9416, *P* < 0.0001. Bland-Altman analyses were performed to evaluate the agreement between immobility- and EEG/EMG-defined sleep in response to treatment with (**G**) vehicle or (**H**) doxepin in 1-h bins over a 24-h cycle. (**G**) Bias 2.5 ± 2.0 min (means ± SD) and 95% limits of agreement from -1.4 to 6.5 min. (**H**) Bias 3.8 ± 2.8 min (means ± SD) with 95% limits of agreement from -1.6 to 9.2 min. EEG, electroencephalogram; EMG, electromyography.

**
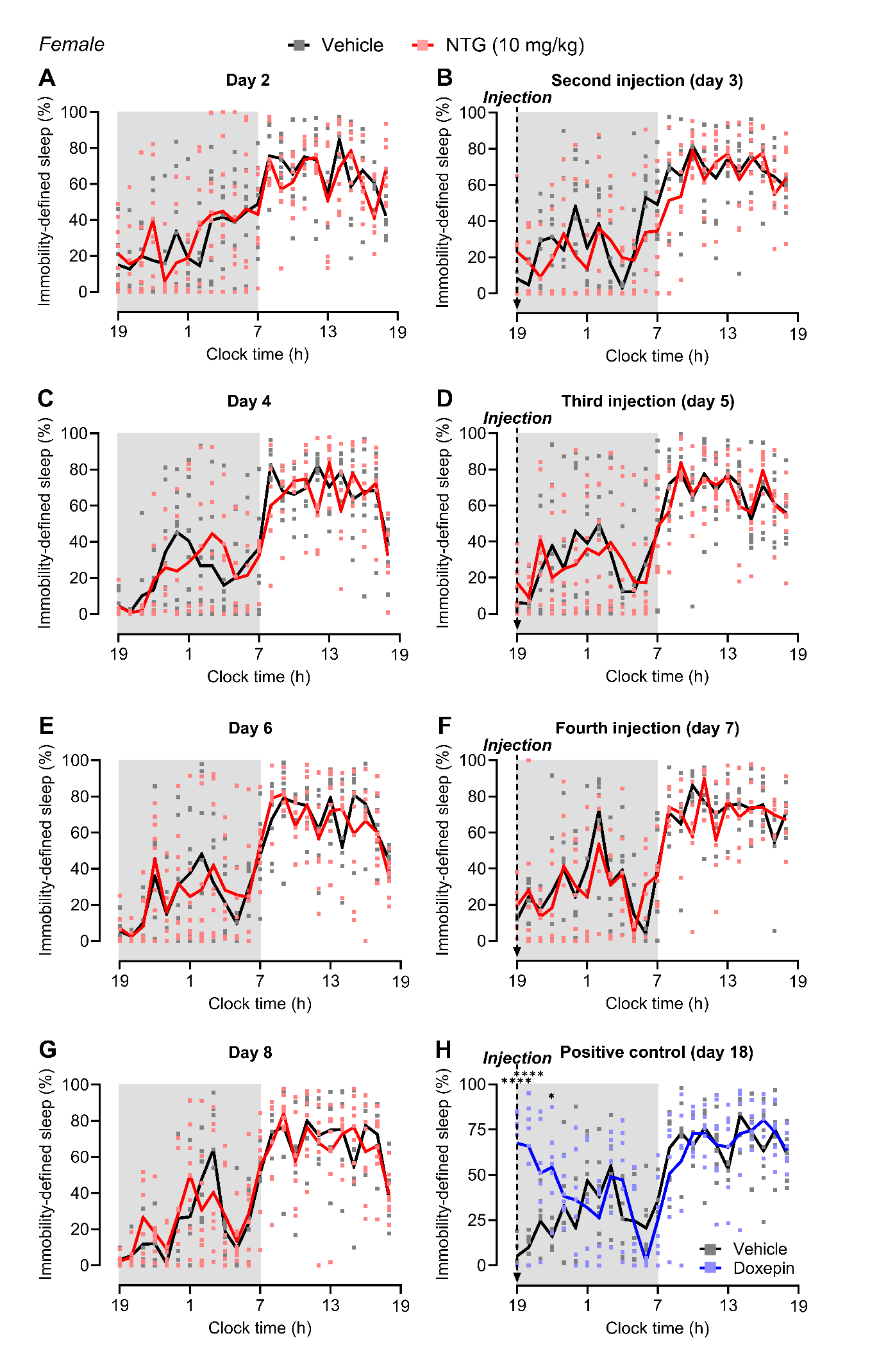
Supplementary Figure 10. NTG-induced chronic migraine-like pain does not affect immobility-defined sleep in female mice.** Female mice received an injection of NTG (10 mg/kg, i.p.) or vehicle every other day for 9 days (five injections in total) at 7 p.m. **(A-G)** The effect of NTG-inducing migraine-like pain on sleep is defined by immobile measurements between days two and eight post-NTG treatment onset, i.e., the period of progression from acute to chronic migraine-like pain. **(H)** Nine days after the last NTG injection (i.e., day 18), mice received a single injection of doxepin (15 mg/kg, i.p.) at 7 p.m. to induce sleep, as a positive control. Control mice were treated with vehicle. Total sleep time is expressed in the percentage of total time in 1-h bins over a 24-h cycle. Arrows indicate the times of injections. The dark phase is shaded in grey. Data were analyzed by two-way repeated-measures ANOVA followed by Sidak’s multiple comparisons when appropriate. (**A**) F(23, 299) = 1.072, *P* = 0.3758, n = 7 mice for vehicle and n = 8 mice for NTG. (**B**) F(23, 299) = 1.144, *P* = 0.2971, n = 8 mice for vehicle and n = 7 mice for NTG . (**C**) F(23, 276) = 1.082, *P* = 0.3652, n = 8 mice for vehicle and n = 6 mice for NTG. (**D**) F(23, 322) = 0.7441, *P* = 0.7987, n = 8 mice for vehicle and n = 8 mice for NTG. (**E**) F(23, 299) = 0.7758, *P* = 0.7611, n = 7 mice for vehicle and n = 8 mice for NTG. (**F**) F(23, 230) = 0.8724, *P* = 0.6357, n = 6 mice for both vehicle and NTG. (**G**) F(23, 276) = 0.9351, *P* = 0.5513, n = 6 mice for vehicle and n = 8 mice for NTG. (**H**) F(23, 276) = 3.769, *P* < 0.0001, n = 7 mice for both vehicle and NTG. F- and *P*-values are shown for interaction factor (treatment and time). **P* < 0.05; *****P* < 0.0001 doxepin vs. vehicle Data values for individual mice are shown as small symbols; lines represent the group means. NTG, nitroglycerin.

**
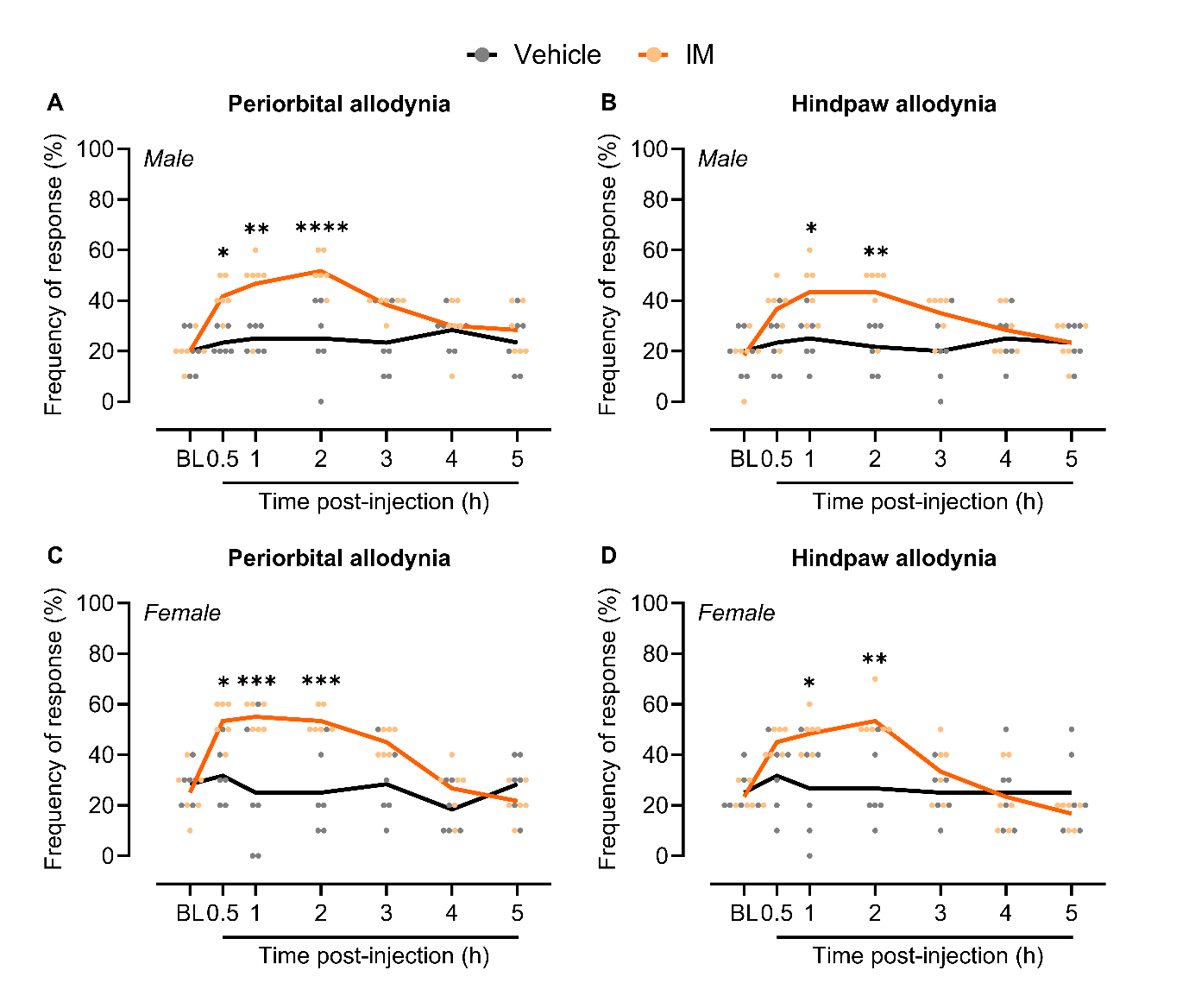
Supplementary Figure 11. Dural IM injection induces robust periorbital and hindpaw allodynia consistent with migraine-like pain in male and female mice.** (**A** and **C**) periorbital and (**B** and **D**) hindpaw frequency of response to tactile stimulation of (**A** and **B**) male and (**C** and **D**) female mice were measured before and over a 5-h period post a single injection of IM mixture or vehicle in the morning. Data were analyzed by two-way repeated-measures ANOVA followed by Sidak’s multiple comparisons. (**A**) F(6, 60) = 6.968, *P* < 0.0001. (**B**) F(6, 60) = 2.969, *P* = 0.0132. (**C**) F(6, 60) = 7.143, *P* < 0.0001. (**D**) F(6, 60) = 6.088, *P* < 0.0001. F- and *P*-values are shown for interaction factor (treatment and time). **P* < 0.05; ***P* < 0.01; ****P* < 0.001; *****P* < 0.0001 IM vs vehicle. Data values for individual mice are shown as small symbols; lines represent the group means; n = 8 mice for all experimental groups. BL, baseline; IM, inflammatory mediators.

**
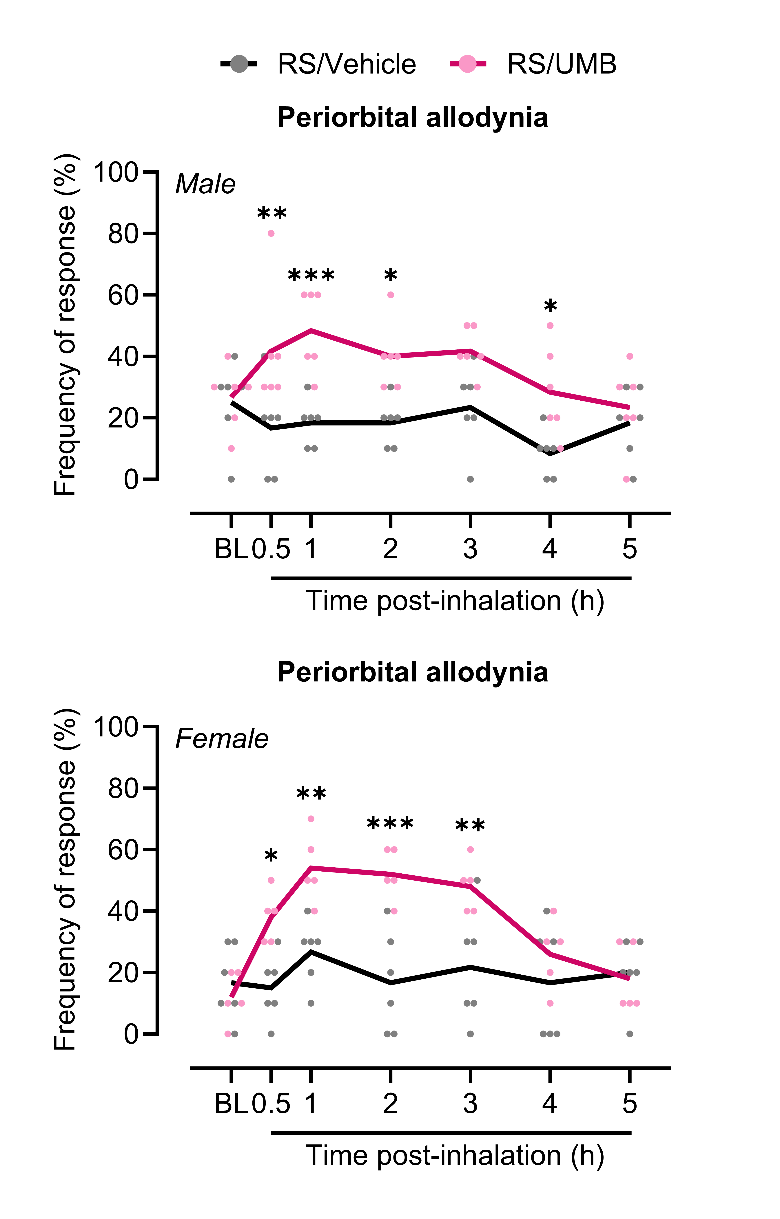
**

**Supplementary Figure 12. UMB inhalation induces robust periorbital allodynia consistent with migraine-like pain in male and female RS-primed mice**. (**A**) male and (**B**) female mice were subjected to repeated RS priming to induce latent sensitization. Sixteen days post-RS priming, periorbital frequency of response to tactile stimulation was measured before (baseline) and over a 5-h period post-UMB or vehicle inhalation in the morning. Data were analyzed by two-way repeated-measures ANOVA followed by Sidak’s multiple comparisons. (**A**) F(1, 10) = 35.01, *P* = 0.0001, treatment factor; n = 6 mice for both RS/UMB and RS/Vehicle groups. (**B**). F(6, 54) = 4.778, *P* = 0.0006, interaction factor (treatment and time); n = 6 mice for RS/UMB and n = 5 mice for RS/Vehicle. **P* < 0.05; ***P* < 0.01; ****P* < 0.001 RS/UMB vs RS/vehicle. Data values for individual mice are shown as small symbols; lines represent the group means. BL, baseline; RS, restraint stress; UMB, umbellulone.


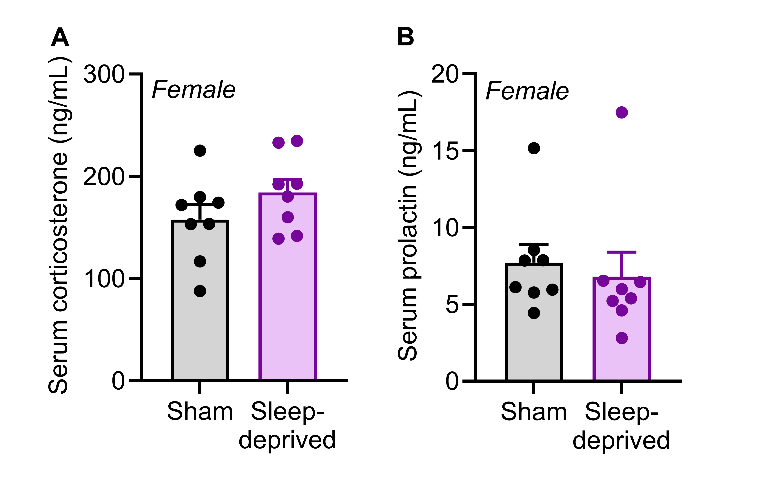


**Supplementary Figure 13. Acute sleep deprivation does not affect serum corticosterone and prolactin levels in female mice.** Female mice were subjected to 6-h sleep deprivation starting at light onset (7 a.m.). Blood was collected at the end of sleep deprivation (1 p.m.) under brief isoflurane anesthesia. Serum (**A**) corticosterone and (**B**) prolactin levels were measured by ELISA. Data were analyzed by (**A**) Student t-test (two-tailed), t(14) = 1.346, *P* = 0.1998 or (**B**) Mann Whitney U-test (two-tailed), *P* = 0.3282. Data are expressed as means ± SEM; n = 8 mice for both sham and sleep-deprived.

**
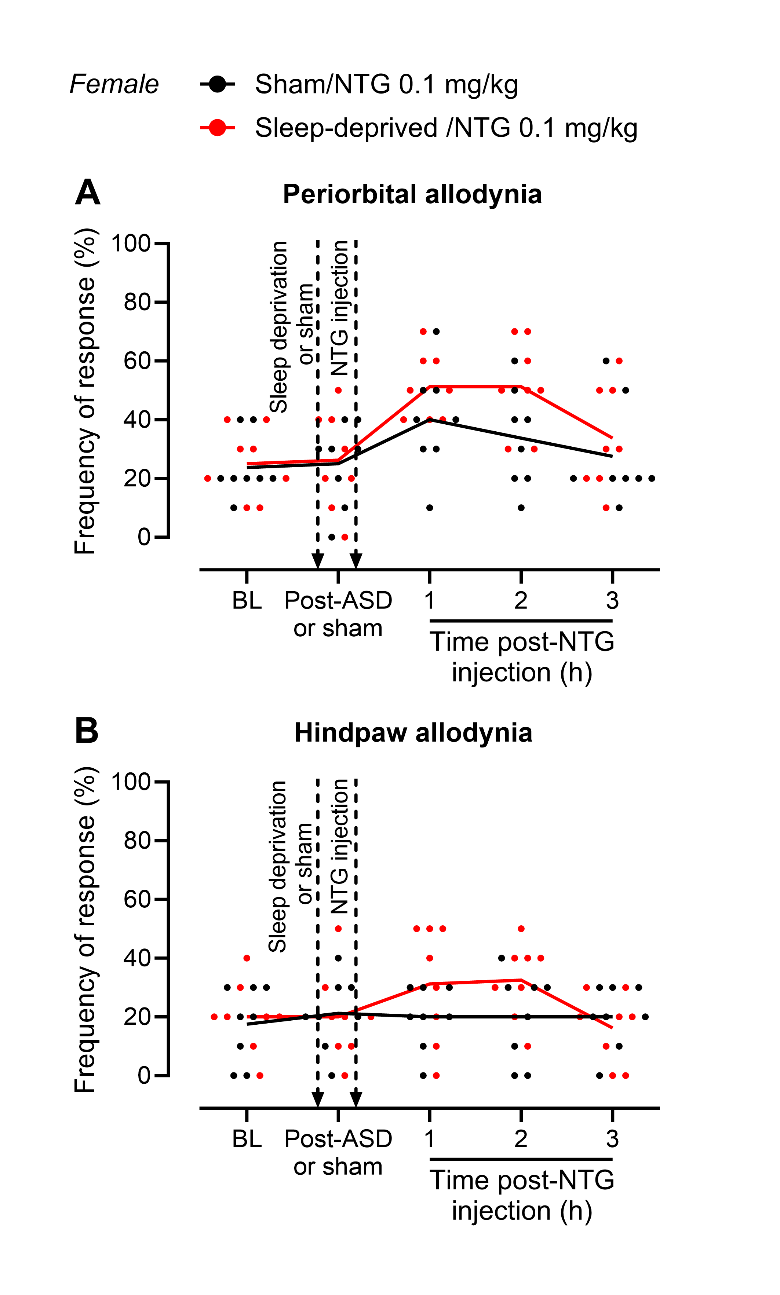
**

**Supplementary Figure 14. Subthreshold dose of NTG induces migraine-like pain in female sleep-deprived mice.** Female mice were subjected to 6-h acute sleep deprivation or sham conditions**. (A)** Periorbital and (**B**) hindpaw frequency of response to tactile stimulation was assessed before (baseline) and immediately after acute sleep deprivation or sham condition. Then, both sleep-deprived and sham mice received a subthreshold dose of NTG (0.1 mg/kg, i.p.) and measurements were performed again hourly over a 3 h period. Cutaneous allodynia was evaluated using von-Frey filaments (0.4 g to the periorbital and 0.6 g to the hindpaw region) and is expressed as a percentage of the frequency of response to tactile stimulation. Data were analyzed by two-way repeated-measures ANOVA. (**A**) F(4, 56) = 1.856, *P* = 0.1310. (**B**) F(4, 56) = 1.775, *P* = 0.1468. F- and *P*-values are shown for interaction factor (treatment and time). Data values for individual mice are shown as small symbols; lines represent the group means; n = 8 for both sham/NTG and sleep-deprived/NTG. ASD, acute sleep deprivation; BL, baseline; NTG, nitroglycerin.

**Supplementary References**

1. Pradhan AA, Smith ML, McGuire B, Tarash I, Evans CJ, Charles A. Characterization of a novel model of chronic migraine. *Pain*. 2014;155(2):269-274.

2. Moye LS, Pradhan AAA. Animal Model of Chronic Migraine-Associated Pain. *Curr Protoc Neurosci*. 2017;80:9 60 1-9 60 9.
